# Identification of atypical T4SS effector proteins mediating bacterial defense

**DOI:** 10.1002/mlf2.12084

**Published:** 2023-09-24

**Authors:** Xi Shen, Zixiang Yang, Zihan Li, Dan Xiong, Jinxing Liao, Weimei He, Danyu Shen, Xiaolong Shao, Ben Niu, Yongxing He, Yong‐Gui Gao, Guoliang Qian

**Affiliations:** ^1^ State Key Laboratory of Biological Interactions and Crop Health, Key Laboratory of Integrated Management of Crop Diseases and Pests, College of Plant Protection Nanjing Agricultural University Nanjing China; ^2^ College of Life Science Northeast Forestry University Harbin China; ^3^ Ministry of Education Key Laboratory of Cell Activities and Stress Adaptations Lanzhou University Lanzhou China; ^4^ School of Biological Sciences Nanyang Technological University Singapore Singapore

**Keywords:** atypical effectors, defense, immunity protein, T4SS, toxic

## Abstract

To remain competitive, proteobacteria use various contact‐dependent weapon systems to defend against microbial competitors. The bacterial‐killing type IV secretion system (T4SS) is one such powerful weapon. It commonly controls the killing/competition between species by secreting the lethal T4SS effector (T4E) proteins carrying conserved XVIPCD domains into competing cells. In this study, we sought knowledge to understand whether the bacterial‐killing T4SS‐producing bacteria encode T4E‐like proteins and further explore their biological functions. To achieve this, we designed a T4E‐guided approach to discover T4E‐like proteins that are designated as atypical T4Es. Initially, this approach required scientists to perform simple BlastP search to identify T4E homologs that lack the XVIPCD domain in the genomes of T4SS‐producing bacteria. These homologous genes were then screened in *Escherichia coli* to identify antibacterial candidates (atypical T4Es) and their neighboring detoxification proteins, followed by testing their gene cotranscription and validating their physical interactions. Using this approach, we did discover two atypical T4E proteins from the plant‐beneficial *Lysobacter enzymogenes* and the phytopathogen *Xanthomonas citri*. We also provided substantial evidence to show that the atypical T4E protein Le1637‐mediated bacterial defense in interspecies interactions between *L. enzymogenes* and its competitors. Therefore, the newly designed T4E‐guided approach holds promise for detecting functional atypical T4E proteins in bacterial cells.

## Impact statement

The bacterial‐killing T4SS functions as a broad contact‐dependent killing device that can transfer toxic T4SS effectors (T4Es) into prey bacterial cells, leading to their death. This study demonstrated the feasibility of discovering previously unidentified atypical T4E proteins that mediate bacterial defense using a T4E‐guided approach, which uncovers special type of T4E/immunity protein pairs and thus highlights the T4SS's diversity. After minor modifications, the T4E‐gudied approach is also applicable for searching atypical effector proteins of the T6SS (type VI secretion system), another antibacterial device shared by a wide range of pathogenic and beneficial bacteria.

## INTRODUCTION

Bacteria establish various interactions with microbial neighbors not only by secreting chemical compounds but also via cell‐to‐cell contacts^1^, ^2^. Through cell‐to‐cell contact, protebacteria deliver toxic effector proteins into competitors, causing their cell death, and hence gains competitive advantages in mixed microbial communities^3^. The type IV secretion system (T4SS) is one such bacterial systems^4^, ^5^, ^6^. T4SS is a multiprotein complex that transports DNA, effectors, and protein–DNA complexes into the extracellular environment or into eukaryotic and prokaryotic target cells^5^, ^7^, ^8^. Based on the physiological functions, T4SS can be classified into three categories: (i) conjugation systems^8^; (ii) effector translocators^9^, ^10^, ^11^, ^12^; and (iii) DNA uptake and release^9^, ^13^. T4SS is also divided into two subgroups, class A (T4ASS) and class B (T4BSS), based on their structural components^14^. T4ASS is mostly used for DNA delivery and is exemplified by the VirB/D4 system of *Agrobacterium tumefaciens* ^15^, ^16^, ^17^. T4BSS is represented by the Dot/Icm system, which is mainly used by intracellular pathogens such as *Legionella pneumophila* to inject effector proteins into eukaryotic hosts to target various immune signaling pathways and promote bacterial infections^18^, ^19^, ^20^.

Recently, the VirB/D4 T4SS in *Lysobacter enzymogenes* and *Xanthomonas citri* was found to be involved in the transfer of lethal T4SS effector (T4E) proteins to bacterial competitors^4^. This functionally distinct T4ASS was defined as the bacterial‐killing T4SS widely present in many bacteria^5^. The characteristic of this bacterial‐killing T4SS is that the *X. citri* VirD4 protein, an ATPase involved in effector recruitment for secretion, interacts with a set of *Xanthomonas* VirD4‐interacting proteins (XVIPs)^4^. In addition, toxic T4Es translocated by the bacterial‐killing T4SSs carry a conserved C‐terminal XVIPCD domain that is required for effector translocation; the toxicities of T4Es in native bacterial cells can be neutralized through protein–protein interactions with adjacent paired immunity proteins^4^. This self‐detoxification mode of T4Es is similar to that of the widespread bacterial toxins of type II toxin–antitoxin (TA) systems, in which intracellular paired antitoxins form protein complexes with toxins to neutralize the antibacterial effects from toxins of type II TA systems^6^.

In the present study, we aimed to understand whether the bacterial‐killing T4SS‐producing bacteria encode atypical T4E proteins that lack of the conserved XVIPCD domain and further investigate their biological functions. Here, we design and verify a simple and efficient T4E‐guided approach capable of discovering such atypical T4E proteins. We also provide a case study showing that Le1637, an atypical T4E protein from the soil antifungal bacterium, *L. enzymogenes* OH11, could mediate bacterial defense during its competition with another soil bacterium, *Enterobacter cloacae* AA4. Our findings reveal that proteobacteria with the bacterial‐killing T4SS could encode atypical T4E proteins mediating bacterial defense, which uncovers a special type of T4E/immunity protein pairs and expands the T4SS's diversity.

## RESULTS

### Workflow of a T4E‐guided approach to find atypical T4E proteins inducing bacterial toxicity

**Figure 1:**
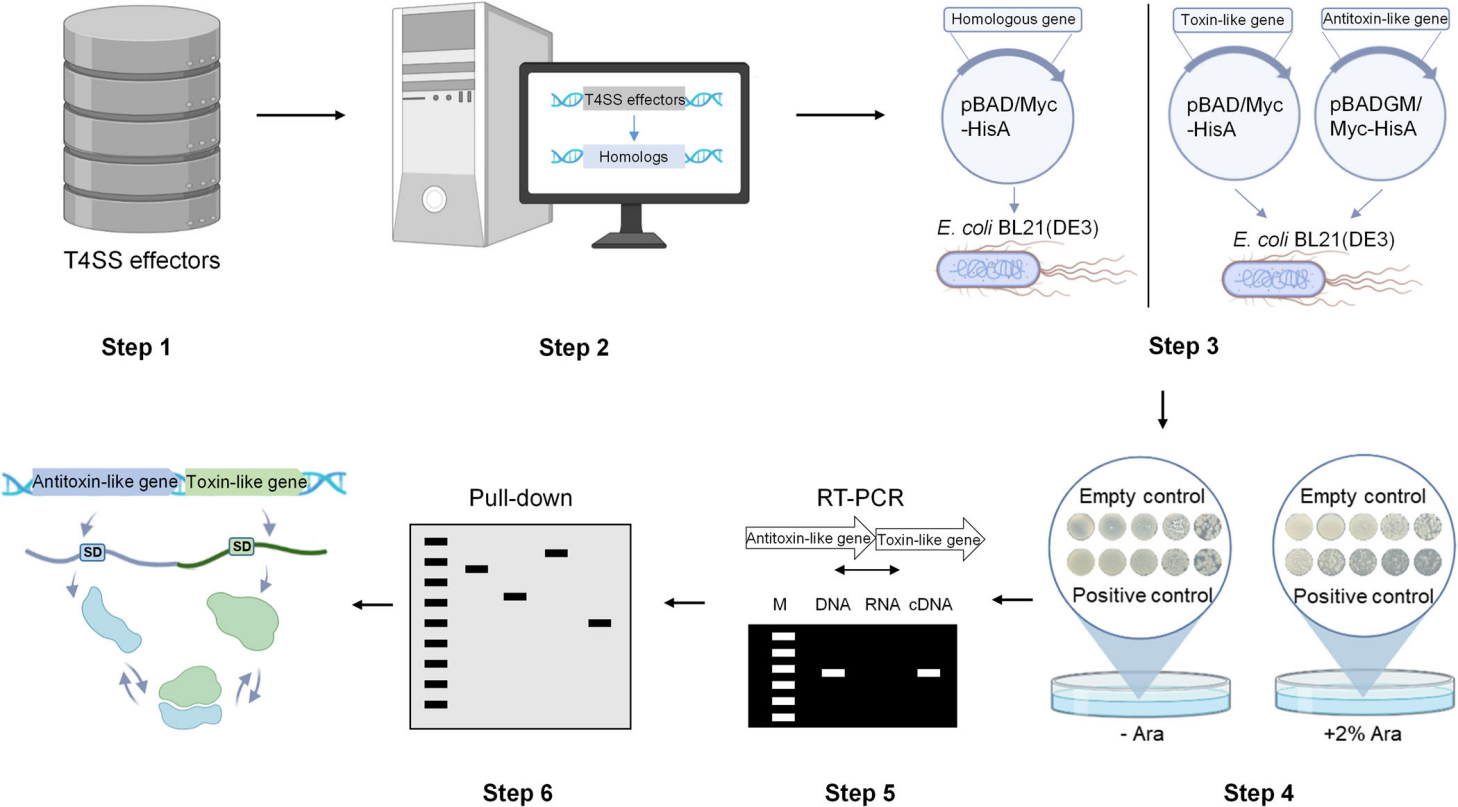
Workflow of the T4SS effector (T4E)‐guided approach to discover atypical T4Es inducing bacterial toxicity in *Escherichia coli*. Online search for T4Es carrying the C‐terminal XVIPCD domain (step 1) and homology comparisons with T4Es to search potential atypical T4E candidates (step 2) were performed. Atypical T4E candidate genes and paired immunity protein‐like genes were cloned into the pBAD/Myc‐HisA vector or pBADGM/Myc‐HisA vector, and cotransformed into *E. coli* BL21 cells. Gene expression was induced in the cytoplasm of *E. coli* driven by an arabinose‐inducible promoter (step 3). These transformed bacterial cells were spotted onto Luria–Bertani agar plates with (+Ara) or without (−Ara) arabinose to observe their growth, which could serve as an indicator to assess the toxicity of a given homolog (step 4). After identifying toxic atypical T4E genes, a genomic mapping analysis was performed to identify potential cotranscribed neighboring genes associated with immunity‐like proteins, followed by testing their gene cotranscription by real‐time PCR (RT‐PCR) (step 5). Finally, a pull‐down assay was used to determine whether the self‐detoxification mechanism of the atypical T4E‐immunity‐like protein relied on direct protein–protein interaction capabilities (step 6).

As previously described, the widespread bacterial‐killing T4SS can translocate toxic T4Es to competing cells, leading to their death. However, the toxicity of T4Es in their native bacterial cells can be neutralized by genome‐adjacent immunity proteins through direct protein–protein interactions^6^. To identify whether the bacterial‐killing T4SS‐producing bacteria could also encode atypical T4Es inducing bacterial toxicity, we designed a simple strategy, defined as a T4E‐guided approach, to identify such atypical T4Es. As shown in Figure 1, the first step of the T4E‐guided approach was to collect a large number of T4Es with a conserved C‐terminal XVIPCD motif from various bacteria with the bacterial‐killing T4SS. These T4Es were then individually searched in their bacterial genomes to identify corresponding homologs without the XVIPCD domain. These XVIPCD domain‐deficient homologs were identified as atypical T4E candidates and subsequently validated experimentally by testing their toxicity in *Escherichia coli*. After identifying toxic atypical T4E genes, genome mapping analyses were performed to identify potential cotranscriptionally adjacent genes of these toxic atypical T4Es. These cotranscribed adjacent genes were then coexpressed with the toxic atypical T4E genes in the *E. coli* cytoplasm, and target genes that could neutralize the atypical T4E‐induced antibacterial toxicity were thought to be paired detoxification proteins. These atypical T4E‐detofication protein pairs were further tested for their protein–protein interaction capabilities.

### Identification of *L. enzymogenes* Le1637 as a previously unknown atypical T4E protein by using the T4E‐guided approach

**Figure 2:**
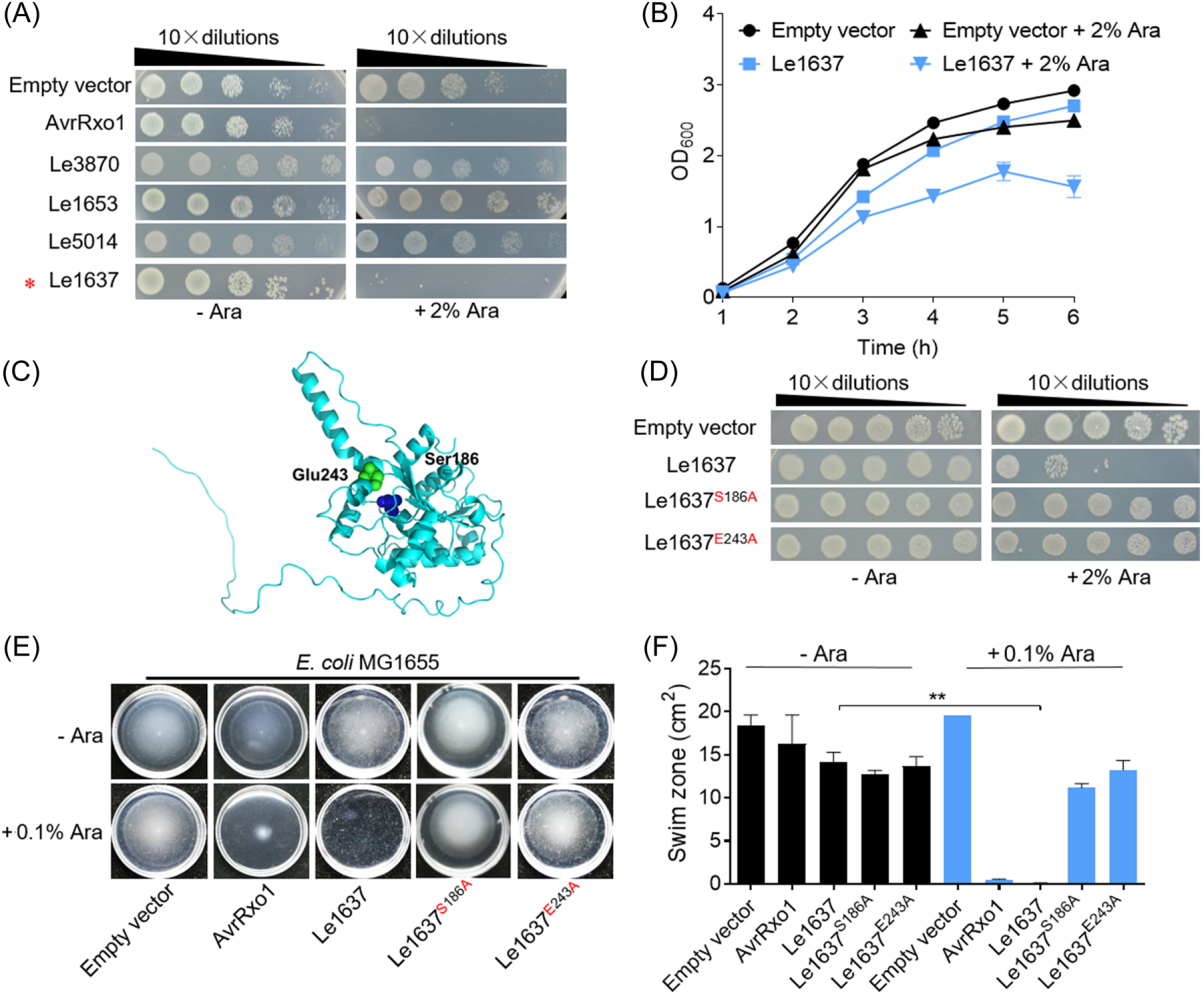
The atypical T4SS effector (T4E), Le1637, from *Lysobacter enzymogenes* OH11 is toxic to *Escherichia coli* cells and its enzymatic activity is important for its toxicity. (A) Expression of the T4E homologous gene in *L. enzymogenes* OH11 under the arabinose‐inducible promoter pBAD/Myc‐HisA vector to assess its toxicity to *E. coli* BL21 (DE3) cells. *E. coli* cells were streaked onto Luria–Bertani medium with (+Ara) or without (−Ara) arabinose added. Plates were incubated at 37°C. The absence of bacterial growth on plates containing arabinose suggested that the gene encoded a toxin. AvrRxo1 was used as a positive control. (B) Growth monitoring of *E. coli* cells carrying Le1637. *E. coli* BL21 (DE3) cultures (OD_600_ = 0.03) transformed with pBAD‐Le1637 and an empty pBAD/Myc‐HisA vector were induced with 2% l‐arabinose at 0 h. Cell growth was recorded hourly at OD_600_. Experiments were repeated at least three times on different days. (C) Homology modeling of Le1637. Two potential residues (S186 and E243) required for Le1637 enzymatic activity were predicted and plotted in a space‐filling model. (D) Replacing S186 and E243 with alanine (A) of Le1637 (designated Le1637^S186A^ and Le1637^E243A^) abolished its induced toxicity to *E. coli* cells. Le1637 and an empty vector were used as positive and negative controls, respectively. Identical results were observed at least three independent times. (E) Induced expression of Le1637 in *E. coli* inhibited swimming motility, but protein variants Le1637^S186A^ and Le1637^E243A^ restored swimming motility. Expression of each given homologous gene was driven by an arabinose‐inducible promoter within the pBAD/Myc‐HisA vector in *E. coli* MG1655. −Ara and +Ara indicate the absence and presence, respectively, of 0.1% l‐arabinose in soft agar (0.35%) plates. Bacteria were incubated at 37°C for 6 h. Motility is indicated by the swimming halos formed on each plate. AvrRxo1 was used as a positive control. Three independent replicates were performed, with similar results. (F) Quantitative swimming activity assays from three experiments. The lengths of the longest axis and the shortest axis of the zones of inhibition were measured and averaged as the radius, *R*. The zones of inhibition were calculated using the formula *π* × *R* ^2^. Mean ± standard deviation of three replicates for each treatment is represented by the column. Asterisks indicate significantly different values according to Student's *t* test (*α* = 0.01).

To test the efficiency of the T4E‐guided approach, we randomly selected T4Es from several bacterial genomes with the bacterial‐killing T4SS as previously described^5^, including *L. enzymogenes* OH11, *X. citri* 306, *Stenotrophomonas maltophilia* K279a, *Lysobacter antibioticus* 76, *L. enzymogenes* C3, *Luteibacter rhizovicinus* DSM16549, and *Neisseria mucosa* C102. By searching their genomes for homologs of each T4E, we indeed found that each searched T4SS‐producing bacterial genome contained one or more homologs corresponding to the respective T4Es (Table S1). Although these T4E homologs contain various predicted domains linked to diverse functions, they all lack the XVIPCD domain, a unique feature shared by all toxic T4Es and responsible for effector translocation^4^. These homologs are defined as atypical T4E candidates. Concurrently, we also predicted the presence of toxins of type II TA systems in *L. enzymogenes* OH11, *X. citri* 306, *S. maltophilia* K279a, *L. enzymogenes* C3, and *L. rhizovicinus* DSM16549 as described above by TADB2^21^ (Figure S1A; Tables S2–S5). However, we did not use TADB2 to predict the presence of toxins of type II TA systems in *L. antibioticus* 76 and *N. mucosa* C102 because their genomes are incomplete and TADB2 requires an intact bacterial genome to function. By comparison, we found that all identified atypical T4E candidates are not the predicted toxins of the type II TA systems. Following the workflow shown in Figure 1, we then screened the antibacterial toxicity of atypical T4E candidates from the plant‐beneficial *L. enzymogenes* OH11, because we recently demonstrated that this bacterium has a functional bacterial‐killing T4SS that mediates soil bacterial competition^22^. As shown in Table S1, although *L. enzymogenes* OH11 encodes 16 T4Es with a conserved C‐terminal XVIPCD domain, we found that only four of them (Le4230, Le0908, Le3432, and Le1288) have homologs lacking the XVIPCD domain and these homologs are Le5014, Le3870, Le1637, and Le1653, respectively (Figure S2), while the remaining 12 T4Es had no detectable homologs using the same bioinformatics approach. We further tested the antibacterial activity of the identified four atypical T4E candidates using a traditional *E. coli*‐based approach^22^, in which the expression of each gene in the cytoplasm was induced by an arabinose‐inducible promoter. As a positive control, expression of the *Xanthomonas oryzae* pv. *oryzae* AvrRxo1^23^ protein with known antibacterial activity in the cytoplasm of *E. coli* MG1655 almost completely inhibited swimming motility. Using this method, we found that when the expression of Le1637 in the cytoplasm of *E. coli* BL21 (OD_600_ = 0.5) was induced by arabinose, it showed potent antibacterial activity, whereas the remaining three candidate genes did not (Figure 2A). On monitoring bacterial growth curves in liquid Luria–Bertani (LB) broth, we found that the expression of Le1637 in the cytoplasm of *E. coli* BL21 significantly reduced the growth ability compared with the *E. coli* BL21 control strain carrying an empty vector, whereas *E. coli* expressing each of the remaining three genes showed similar growth to the control strain (Figures 2B and S3). Sequence analysis showed that Le1637 was a member of phospholipase A1 protein distributed in multiple bacterial genomes (Table S6).

To test whether the predicted enzymatic activity of Le1637 correlates with its antibacterial activity, we carried out a homology modeling analysis, which revealed that Le1637 has two residues, S186 and E243, which were predicted to be essential for Le1637 enzymatic activity (Figure 2C). We thus replaced S186 and E243 with alanine (A), respectively, used site‐direct mutagenesis to generate two protein variants, Le1637^S186A^ and Le1637^E243A^. As expected, arabinose‐induced expression of the *Le1637* ^*S186A*^ or *Le1637* ^*E243A*^ variant gene in *E. coli* BL21 cytoplasm failed to inhibit bacterial growth (Figure 2D). To better visualize the antibacterial toxicity of Le1637 expression, we next established a bacterial swimming‐indicator method^24^. We hypothesized that if the growth of *E. coli* was inhibited by Le1637, then the swimming motility of *E. coli* expressing Le1637 would in principle be inhibited or abolished. To achieve this goal, we chose *E. coli* MG1655 because this strain is known to produce enhanced motility on agar plates, as previously described^25^. We found that arabinose‐induced expression of Le1637 in the cytoplasm of MG1655 significantly reduced the swimming zone. Differentially, arabinose‐induced expression of two Le1637 variants, Le1637^S186A^ and Le1637^E243A^, in the cytoplasm of MG1655 did not cause reduce swimming zone (Figure 2E,F). These results provide a line of experimental evidence supporting the effectiveness of the T4E‐guided approach for the discovery of atypical T4E proteins.

**Figure 3:**
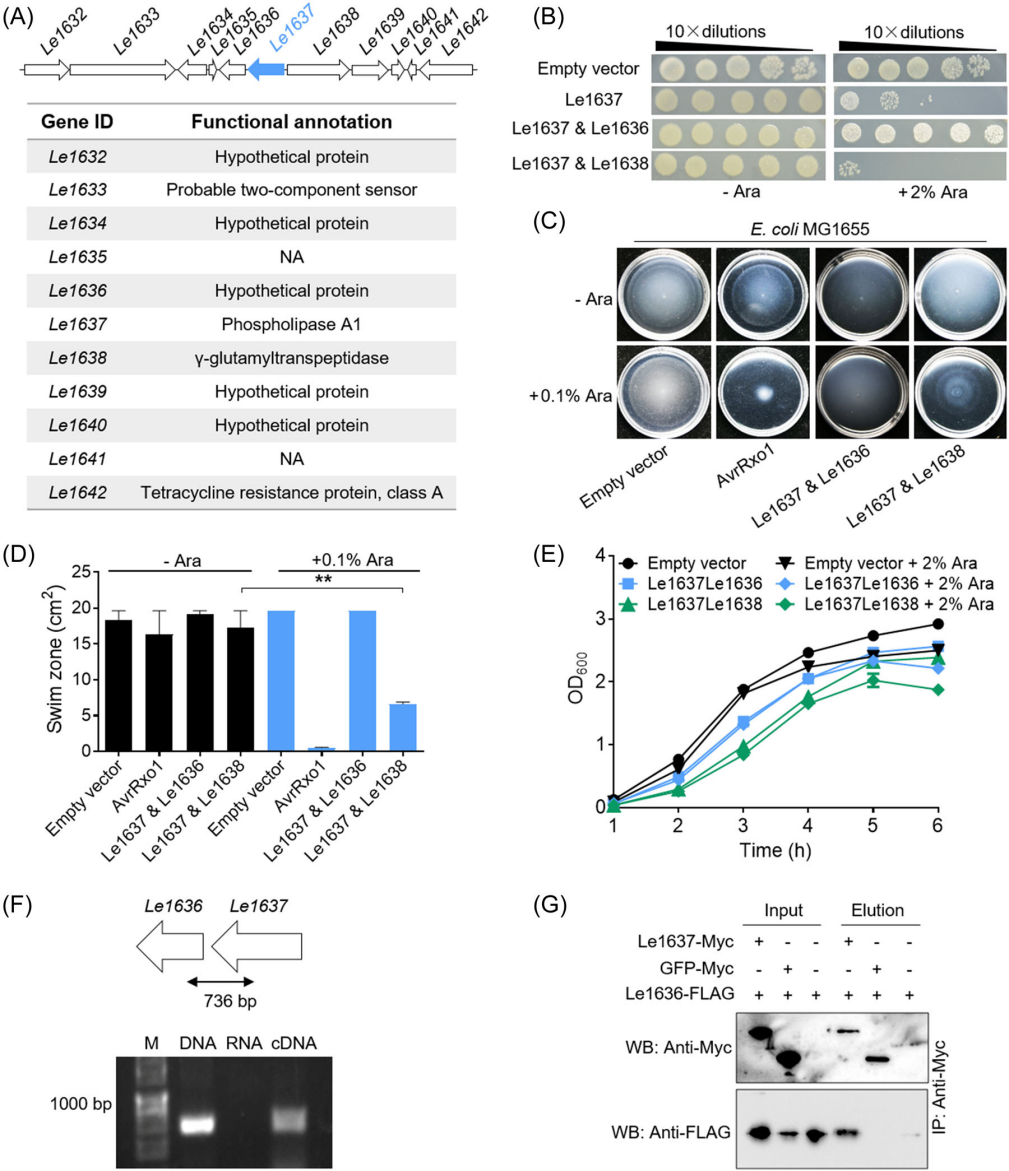
The atypical T4SS effector (T4E), Le1637, has a paired immunity‐like protein Le1636. (A) The genomic organization of *Le1637* is highlighted in blue. (B) Toxicity of Le1637 to *Escherichia coli* BL21 was neutralized by coexpression of Le1636 but not Le1638. (C) Induced coexpression of Le1637 and Le1636 in *E. coli* restored motility. *E. coli* MG1655 coexpressing Le1637‐Le1636 or Le1637‐Le1638 from an arabinose‐inducible promoter. AvrRxo1 was used as a positive control. Three independent replicates were performed, with similar results. (D) Quantitative swimming activity assays from three experiments. Mean ± standard deviation of three replicates for each treatment is represented by the column. Asterisks indicate significantly different values according to Student's *t* test (*α* = 0.01). (E) Growth inhibition of *E. coli* cells triggered by Le1637 was restored by coexpressing with Le1636 but not Le1638. (F) Real‐time PCR assay revealed that Le1637 was cotranscribed with Le1636. M, Marker; DNA (genomic DNA of OH11), positive control; RNA, negative control. (G) Pull‐down assay revealed that Le1637‐Myc directly interacted with Le1636‐FLAG. Myc fusion proteins were pulled down to react with antibody.

The above findings prompted us to address whether Le1637 acts in a manner similar to that of T4Es. To achieve this goal, we first searched for paired immunity‐like protein candidates of Le1637, because toxic T4E genes are known to cotranscribe with paired immunity protein genes, and T4Es‐paired immunity proteins can form protein complexes for self‐detoxification^26^. Since Le1637 is a homolog of the T4E Le3432, we thus reasonably attempted to identify the Le1637's paired immunity‐like protein candidate from its genomic neighbors. As shown in Figure 3A, *Le1636* and *Le1638* are two neighboring protein genes of Le1637, encoding a hypothetical protein and γ‐glutamyltranspeptidase, respectively. To test whether they are potential paired immunity‐like proteins of Le1637, we coexpressed Le1636 or Le1638 with Le1637 on different plasmids in the cytoplasm of *E. coli* BL21, and found that the antibacterial toxicity of Le1637 was neutralized by arabinose‐induced coexpression of Le1636, but not Le1638 (Figure 3B). Using the *E. coli* MG1655‐dependent swimming approach and growth monitoring, we further observed that Le1637‐triggered swimming and growth inhibition in MG1655 and BL21, respectively, could be fully rescued by coexpressing with Le1636 but not Le1638 (Figure 3C–E). Therefore, Le1636 most likely acted as an immunity‐like protein for Le1637. By real‐time PCR (RT‐PCR), we also demonstrated that the *Le1637‐Le1636* gene pair was cotranscribed (Figure 3F). Using in vitro pull‐down assays, we further found that Le1636‐FLAG directly bound to Le1637‐Myc (Figure 3G). Collectively, these findings revealed that Le1637 is a toxic atypical T4E protein.

### Le1637‐mediated *L. enzymogenes* defense in bacterial contact‐dependent interspecies interactions

**Figure 4:**
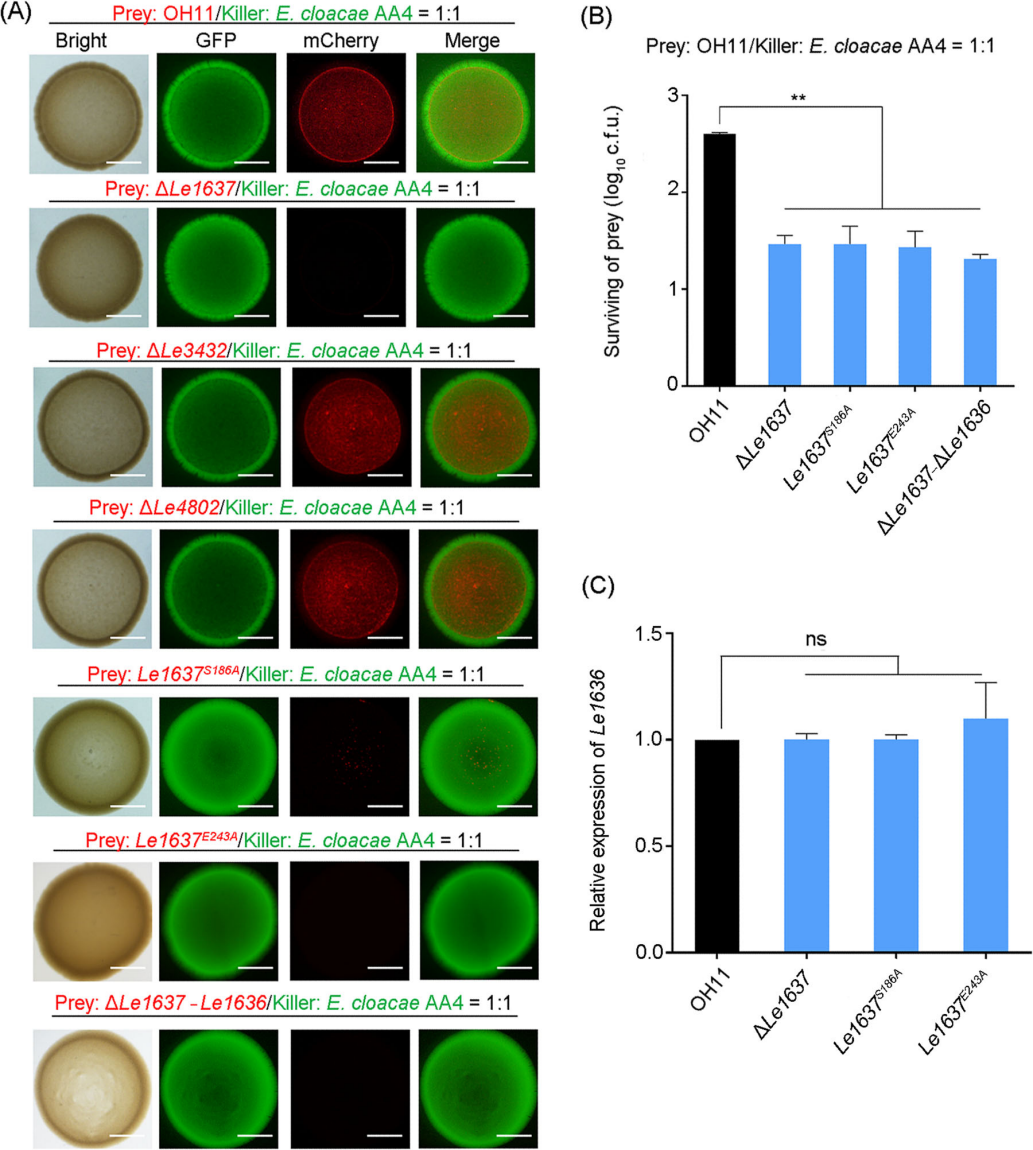
Le1637‐mediated bacterial defense during cell‐to‐cell contact between *Lysobacter enzymogenes* and its competitor. (A) Contact‐dependent defense activity of mCherry‐labeled OH11 against GFP‐labeled *Enterobacter cloacae* AA4. mCherry‐labeled prey strains (OH11, Δ*Le1637*, Δ*Le3432*, Δ*Le4802*, *Le1637* ^*S186A*^, *Le1637* ^*E243A*^, and Δ*Le1637*‐Δ*1636*) were mixed with GFP‐labeled *E. cloacae* AA4 at a ratio of 1:1 and coinoculated on agar plates for 24 h, followed by observation of the GFP and mCherry fluorescence signals. Le3432, a predicted T4SS effector, is a Le1637 homolog; Le4802, a predicted and experimentally validated toxin of type II toxin–antitoxin systems by using the online database TADB2. Le1637^S186A^ and Le1637^E243A^, protein variants via point mutations on the chromosome. Bars represent 2 mm. (B) Quantification of living prey cells under co‐culture conditions. Mean ± standard deviation (SD) of three replicates for each treatment is represented by the column. Asterisks indicate significantly different values according to Student's *t* test (*α* = 0.01). (C) Deletion of *Le1637* and point mutations of S186A and E243A of Le1637 on chromosome did not affect the expression of *Le1636*, as measured by qRT‐PCR. Mean data ± SD from triplicate experiments are presented. GFP, green fluorescent protein; ns, no significant changes.

What is the function of Le1637 acting as an atypical T4E in the T4SS‐producing, plant‐beneficial *L. enzymogenes* OH11? To address this question, we first generated an in‐frame *Le1637* deletion mutant in the wild‐type OH11 background. As a natural predator of fungi, *L. enzymogenes* OH11 can use type IV pilus‐driven twitching motility to move toward nearby fungi and inhibit fungal growth by secreting an antibiotic called the heat‐stable antifungal factor, HSAF^27^. We, therefore, tested whether Le1637 plays a role in these two key features of biocontrol. Our results showed that the mutation of *Le1637* did not impair twitching motility and HSAF production (Figure S4). We then investigated whether Le1637 is involved in T4SS‐mediated contact‐dependent bacterial killing, which has been reported to be a novel antibacterial mechanism independent of antibacterial metabolites^22^. To test this hypothesis, both the wild‐type and the *Le1637* deletion mutant of OH11 were labeled with mCherry and served as killer strains. The model strain *E. coli* BL21 and two soil‐borne bacteria (*Pseudomonas fluorescens* 2P24 and *Pseudomonas protegens* Pf‐5) that share similar ecological niches with OH11 were labeled by green fluorescent protein (GFP) and regarded as prey strains. All three prey strains could be killed by OH11 using T4SS as described in our laboratory^22^. We coinoculated the mCherry‐labeled OH11 or Δ*Le1637* strain with each of the three GFP‐labeled prey strains at a ratio of 1:1 on agar plates to mimic their cell‐to‐cell contact behavior as previously described^22^. When using GFP or mCherry signal intensity as the indicator of bacterial growth in coinoculated colonies, we found that Δ*Le1637* showed visible killing efficiencies against all three tested prey strains similar to wild‐type OH11. This suggested that Le1637 did not seem to be involved in T4SS‐mediated bacterial killing events (Figure S5). Finally, we tested whether Le1637 is involved in OH11 defense against contact‐dependent attack by other soil‐borne bacteria. We selected a variety of soil‐borne bacteria available in the laboratory and found that *E. cloacae* AA4^28^ could efficiently kill OH11 via cell‐to‐cell contact. We coinoculated GFP‐labeled *E. cloacae* AA4 with mCherry‐labeled OH11 or Δ*Le1637* at a ratio of 1:1 on agar plates and found that the mutation of *Le1637* significantly attenuated the ability of *L. enzymogenes* OH11 to defend against *E. cloacae* AA4 attack (Figure 4A,B). This role of Le1637 in *L. enzymogenes* appeared to be specific, as in the wild‐type OH11 background, mutations of *Le3432*, a predicted T4E of the Le1637 homolog, or *Le4802*, a predicted and experimentally validated toxin of the type II TA system (Figure S1B), did not significantly alter the ability of *L. enzymogenes* to defend against the intercellular attack of *E. cloacae* AA4 (Figure 4A). Furthermore, we found that Le1637‐GFP was distributed throughout the cells when applied to OH11 monoculture on agar plates. This cellular localization was not changed when OH11 and AA4 were cocultured on agar plates to mimic their cell‐to‐cell contacts (Figure S6). Moreover, no killing activity was observed when the growth of wild‐type OH11 and AA4 was separated by a 0.22‐μm filter (Figure S7). These findings revealed that the presence of Le1637, an atypical T4E, enabled *L. enzymogenes* to efficiently defend against ecological competitors via cell‐to‐cell contact.

To support the above findings, we further applied two Le1637 toxicity‐deficient mutants (*Le1637* ^*S186A*^ and *Le1637* ^*E243A*^) with chromosomal site mutations. We found that both point mutants showed a similar phenotype to the *Le1637* in‐frame deletion mutant in defense against challenge by a competitor (AA4) (Figure 4A,B). We also generated a double mutant (Δ*Le1637*‐*Le1636*) and found that this double mutant strain also lost its defense against AA4 attack (Figure 4A,B).

To demonstrate that the function of Le1637 is not due to its effect on the transcript level of *Le1636*, we tested the transcript levels of *Le1636* in the wild‐type strain OH11, Δ*Le1637*‐*Le1636*, *Le1637* ^*S186A*^, and *Le1637* ^*E243A*^. There were no significant differences in *Le1636* transcript levels between wild‐type OH11 and all tested mutants (Figure 4C). These results collectively supported the conclusion that the atypical T4E, Le1637, mediated the defense of *L. enzymogenes* against competitor challenge.

### T4E‐guided approach enabled the discovery of *X. citri* XAC2247 as an additional atypical T4E protein

**Figure 5:**
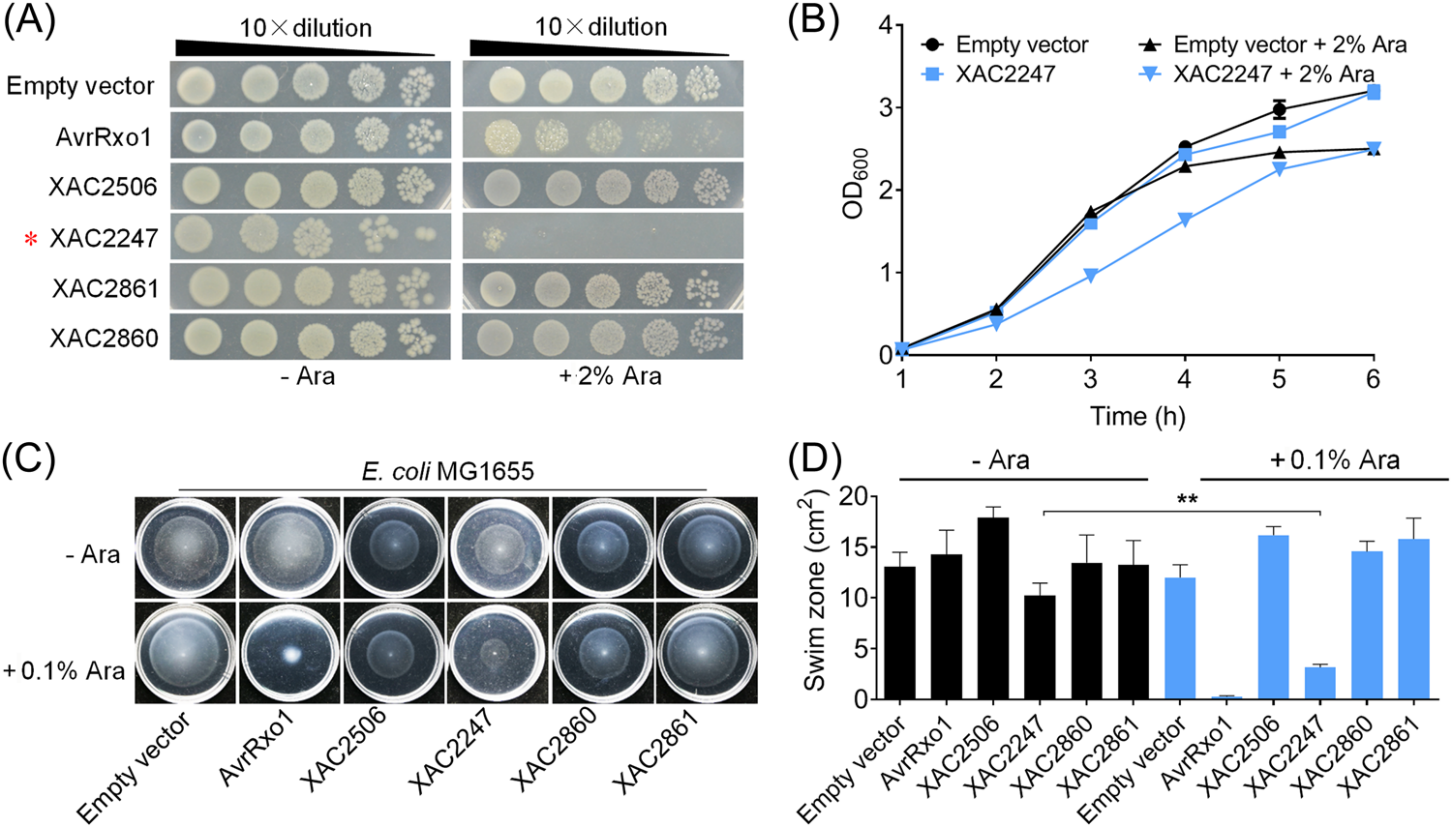
The *Xanthomonas citri* atypical T4SS effector, XAC2247, is toxic to *Escherichia coli*. (A) Expression of homologous genes from *X. citri* 306 under the arabinose‐inducible promoter pBAD/Myc‐HisA vector was conducted to assess their toxicity to *E. coli* BL21 (DE3) cells. *E. coli* cells were streaked onto Luria–Bertani medium supplemented with (+Ara) or without (−Ara) arabinose. Plates were incubated at 37°C. (B) Growth monitoring of *E. coli* cells carrying XAC2247. *E. coli* BL21 (DE3) cultures (OD_600_ = 0.03) transformed with pBAD‐XAC2247 and an empty pBAD/Myc‐HisA vector were induced with 2% l‐arabinose at 0 h. Cell growth was recorded hourly at OD_600_. Experiments were repeated at least three times on different days. (C) Induced expression of XAC2247 in *E. coli* inhibited swimming motility. AvrRxo1^29^ was used as a positive control. Three independent replicates were performed, with similar results. (D) Quantitative swimming activity tests from three experiments. Mean ± standard deviation of three replicates for each treatment is represented by the column. Asterisks indicate significantly different values according to Student's *t* test (*α* = 0.01).

To test whether the T4E‐guided approach has broad application potential, we switched the working model from plant‐beneficial bacteria to phytopathogen bacteria. To achieve this, we selected the phytopathogen *X. citri* 306 as an additional model, because this bacterium was first reported to carry a bacterial‐killing T4SS^4^. Among the 16 T4Es, only one (XAC3266) was predicted to have the corresponding homologous protein (out of five in total) in the genome of *X. citri* 306 (Table S1). Each homologous gene was amplified by PCR and cloned into the pBAD/Myc‐HisA vector, then introduced into *E. coli* BL21, and expressed in the cytoplasm of *E. coli* driven by an arabinose‐inducible promoter within the plasmid. This screen did further lead to the discovery of XAC2247, but not the remaining four genes, as an additional toxin‐like protein with antibacterial activity (Figure 5A,B). While XAC2247 was also distributed in many bacterial genomes (Table S7), it is also not predicted as a toxin of type II TA system. Using *E. coli* MG1655‐dependent swimming as an additional indicator, we also observed that none of the four remaining homologous proteins of XAC3266 inhibited bacterial swimming motility, whereas XAC2247 had a strong effect on its expression in the cytoplasm of MG1655, as did the positive control gene, *avrRxo1* (Figure 5C,D). Collectively, these results suggested that XAC2247 most likely functioned as an atypical toxic T4E.

**Figure 6:**
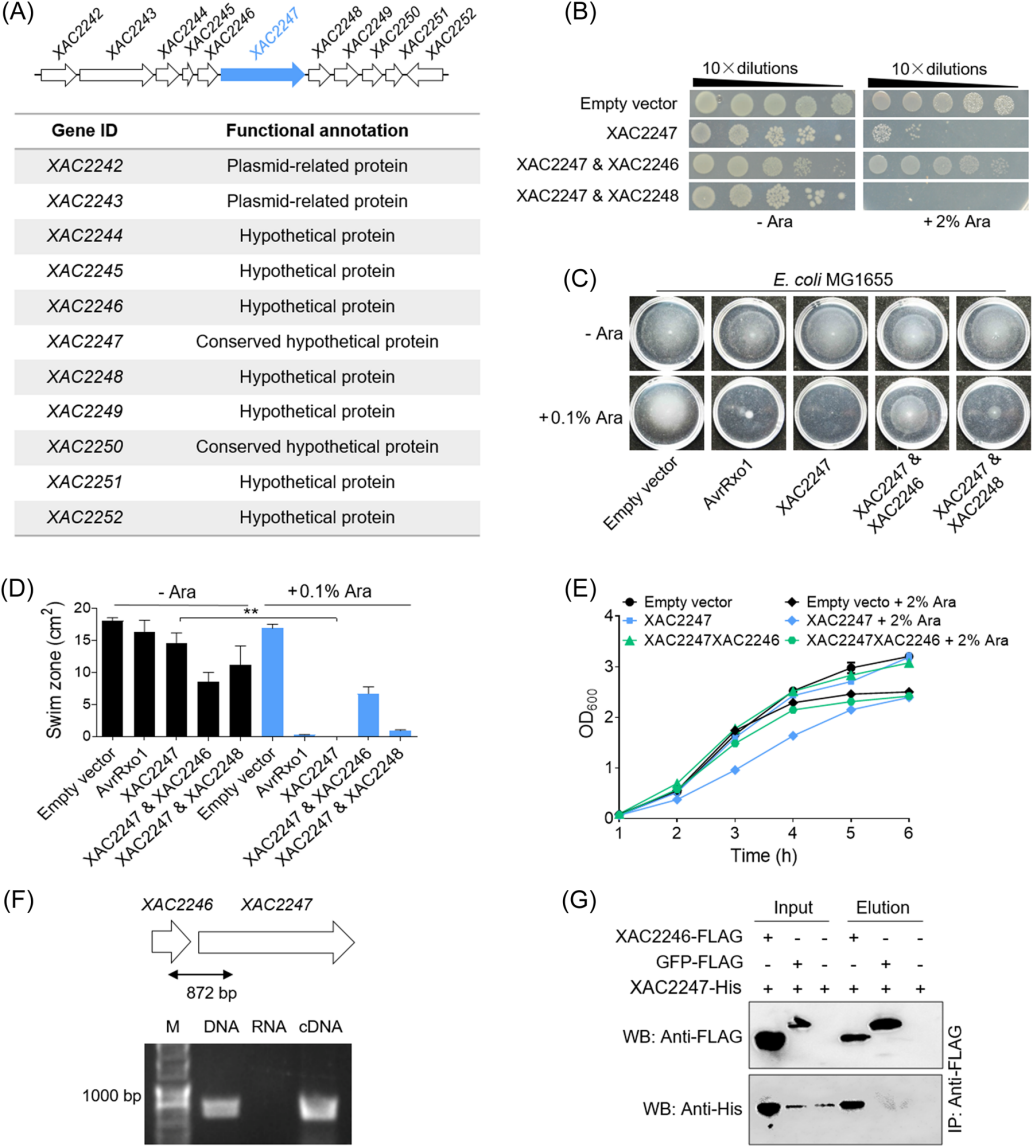
XAC2247 has a paired immunity‐like protein XAC2246. (A) Genomic organization of the toxin XAC2247, highlighted in blue. (B) XAC2247 and XAC2246 formed an atypical T4SS effector‐immunity‐like protein pair. Toxicity of XAC2247 to *Escherichia coli* BL21 was neutralized by coexpression of XAC2246 but not XAC2248. (C) Induced coexpression of XAC2247 and XAC2246 in *E. coli* rescued swimming motility. XAC2247‐XAC2246 and XAC2247‐XAC2248 were coexpressed from an arabinose‐inducible promoter in *E. coli* MG1655. AvrRxo1 was used as a positive control. Three independent replicates were performed, with similar results. (D) Quantitative swimming activity tests from three experiments. Mean ± standard deviation of triplicate replicates for each treatment is represented by the column. Asterisks indicate significantly different values according to Student's *t* test (*α* = 0.01); (E) growth inhibition of *E. coli* cells triggered by XAC2247 was rescued by coexpression with XAC2246 but not XAC2248. (F) Real‐time PCR assay revealed cotranscription of *XAC2247* with *XAC2246*. M, marker; DNA (genomic DNA of *Xanthomonas citri* 306), a positive control; RNA, a negative control. (G) Pull‐down assay revealed that XAC2247‐His directly interacted with XAC2246‐FLAG. The FLAG‐fused proteins were pulled down after reacting with antibodies.

Next, we provided several lines of experimental evidence supporting the above findings. First, we found that XAC2246 is an adjacent paired immunity‐like protein of XAC2247 (Figure 6A), as arabinose‐induced coexpression of XAC2246, but not XAC2248, was found to completely neutralize the antibacterial toxicity elicited by XAC2247 in the cytoplasm of *E. coli* BL21 (Figure 6B). Similar findings were observed when this coexpression was switched to growth monitoring and MG1655‐dependent swimming assays in liquid LB broth (Figure 6C–E). RT‐PCR results supported cotranscription of the *XAC2247*‐*XAC2246* gene pair (Figure 6F). Pull‐down experiments showed that XAC2247‐His could directly bind to XAC2246‐FLAG (Figure 6G).

## DISCUSSION

In the study, we presented a simple yet robust approach, termed the T4E‐guided approach, for the discovery of atypical T4E proteins. The simplicity of this approach makes it suitable for use by bacteriologists, as it only requires a BlastP search to be performed to identify T4E protein homologs in target T4SS‐producing bacteria that are ubiquitous in the genomes. The strength of this strategy is that we indeed identified many atypical T4E proteins and further identified two previously unidentified but experimentally validated atypical T4E‐like proteins: Le1637 and XAC2247. Le1637 and XAC2247 could bind to their respective neighbor immune‐like proteins to detoxify their toxicity in *E. coli* through protein–protein interactions. This toxin‐detoxification mode expressed by the atypical T4E/detoxification protein pairs is similar to that of the canonical T4E/immunity protein pairs, which uncovers special types of T4E/immunity protein pairs and thus expands the diversity of the bacterial‐killing T4SS. It is also noteworthy that while both the atypical T4E proteins, Le1637 and XAC2247, are not predicted as toxins of the bacterial type II TA systems, their detoxification pattern of binding to neighbor proteins is also similar to that of the TA pairs of the type II TA systems, providing a clue to an evolutionarily link between atypical T4E proteins with toxins of the type II TA systems.

While Le1637‐Le1636 and XAC2247‐XAC2246 are not predicted to be a TA pair of the type II TA system, their discovery as atypical T4E/immunity protein pairs, bearing certain similarities in detoxification modes and cotranscription to the type II TA systems, suggests that the identification of the atypical T4Es, Le1637 and XAC2247, might help bacteriologists to uncover previously unknown functions of special toxins. According to earlier studies, canonical toxins can be encoded on plasmids or bacterial genomes and are mainly involved in postsegregational killing^29^, phage‐abortive infection^30^, and persistence^31^. While we did not know the function of XAC2247 defined as a hypothetical protein, in the current study, it was found that Le1637 was involved in a hitherto unreported bacterial defense. This provides a case study pointing to the biological/ecological significance of atypical T4E proteins. While we did not currently know why Le1637 acquired such a key role, its discovery might provide clues. As previously mentioned, Le1637 is a homolog of the T4E Le3432, and was thought to be injected into competing cells to promote the contact‐dependent killing ability of *L. enzymogenes* using T4SS. This led us to propose that the purpose of Le1637 as the genomic homolog of Le3432 might be to help *L. enzymogenes* gain a survival advantage in the bacterial interspecies war. In this way, Le3432 acted as a “gunshot” of the T4SS killing device, attacking its susceptible competitors, while Le1637 functioned as a molecular “shield,” protecting *L. enzymogenes* cells from attack by other neighboring bacterial species such as *E. cloacae* AA4. Understanding the mechanism by which Le1637 mediates bacterial defense will answer this intriguing question in the future.

In conclusion, the present study demonstrates that it is feasible to discover previously unidentified atypical T4E proteins by using a T4E‐guided approach. This can efficiently enable the transition of T4Es from being the subject of mechanistic investigations to technical applications. We propose that the developed T4E‐guided approach has broad applications, with only slight modifications. For example, it is believed that scientists could find potentially toxic proteins by searching for atypical T6SS effectors, another widespread bactericidal device shared by a wide range of pathogenic and beneficial bacteria^32^.

## MATERIALS AND METHODS

### Bacterial strains, plasmids, and growth conditions

The bacterial strains and plasmids used in this study are presented in Table S8. *L. enzymogenes* strain OH11 (CGMCC No. 1978) and *X. citri* 306 (NC_003919.1) were grown in LB at 28°C using the following antibiotics: kanamycin (Km), 30 μg/ml, and gentamicin (Gm), 150 μg/ml for plasmid maintenance. *E. coli* BL21, *E. coli* MG1655, and *E. cloacae* AA4 strains were grown in LB medium at 37°C, whereas *P. fluorescens* 2P24 and *P. protegens* Pf‐5 were grown in the same medium at 28°C.

### Bioinformatics analyses

The T4SS structural proteins of the phylogenetically related bacterial strain *X. citri* 306 (NC_003919.1) were used as queries in a local BlastP search to identify the corresponding homologs in the *L. enzymogenes* OH11 genome. A protein was considered to be present when the *E* value was below 10^−5^ and the percentage similarity to the *X. citri* 306 homologous protein was above 35%. To predict the occurrence of XVIPCD‐domain proteins in OH11, the XVIPCD domain sequences of 13 *X. citri* XVIPCD proteins derived from *X. citri* 306^4^ were aligned using the MUSCLE tool, and a Hidden Markov Model (HMM) profile was constructed. An HMM search was then performed against the OH11 proteins using the HMM search program implemented in HMMER^33^. An XVIPCD domain was considered to exist when the HMM search *E* value was below 10^−5^. The T4Es from bacterial strains other than OH11 were collected from an earlier reference^5^.

### Online search for homologous proteins of T4Es in various T4SS‐producing bacteria

Bacterial‐killing T4Es have been predicted in a variety of bacterial species^5^. In this study, we selected seven strains of T4SS‐producing bacteria, including *L. enzymogenes* OH11 (CGMCC No. 1978), *X. citri* 306 (NC_003919.1), *S. maltophilia* K279a (GCF_000072485.1), *L. antibioticus* 76 (GCA_001442745.1), *L. enzymogenes* C3 (CP013140), *L. rhizovicinus* DSM16549 (CP017480), and *N. mucosa* C102 (GCA_000186165). We searched the genome of the respective bacterial host for each predicted T4E to identify potential homologs by running local BlastP. Homologous protein was considered to be present when the *E* value was below 10^−5^ and the percentage similarity to the corresponding T4E protein was above 20%.

### Genetic methods

A double‐crossover homologous recombination approach was used to generate in‐frame deletion mutants of OH11 as previously described^34^. Briefly, 300‐ to 500‐bp DNA fragments from the two flanking gene regions were cloned into the broad host suicide vector pEX18Gm after amplification by PCR using specific primers. The recombinant vectors were introduced into OH11 cells by electroporation. Single‐crossover recombinants in LB medium supplemented with Km (25 μg/ml) and Gm (150 μg/ml) in 1.2% LB agar (LA) were chosen. Transformants were then grown in LA Petri dishes containing Km (100 μg/ml) and 10% (wt./vol.) sucrose to select for double crossovers. Mutations were confirmed by PCR using specific primers.

### Homology modeling of Le1637

Homology modeling of Le1637 was carried out using the AlphaFold2^35^ program (https://colab.research.google.com/github/sokrypton/ColabFold/blob/main/AlphaFold2.ipynb). The Uniprot^36^ program (https://www.uniprot.org/) was then used to further search the homologs of the modeled structures to identify potential enzyme active sites.

### Detection of atypical T4E‐induced toxicity in *E. coli* BL21

Each T4E homolog gene was cloned into the arabinose‐inducible plasmid pBAD/Myc‐HisA (ampicillin resistant), as previously described^37^. As negative and positive controls, the empty vector and the *avrRxo1* gene with known antibacterial activity from *X. oryzae* pv. *oryzae* RS105 were utilized^23^, respectively. Each recombinant construct was electroporated individually into *E. coli* BL21 (DE3). All transformed strains were grown in LB medium without arabinose. Exponentially growing cells were harvested and adjusted to an optical density of 0.5 at 600 nm (OD_600_) and serially diluted (×10) with fresh LB. The final step involved spotting 5 μl of each bacterial dilution culture onto the surface of an LA dish that had been modified with or without 2% arabinose. Using a Nikon camera, LA dishes were incubated at 37°C for 24 h (D7100). A similar procedure was used to examine the growth of *E. coli* strains with atypical T4E or immunity‐like protein genes. According to an earlier study in our laboratory^24^, the T4E homolog gene was cloned into the pBAD/Myc‐HisA plasmid and the immunity‐like protein gene was cloned into a modified pBADGM/Myc‐HisA (Gm‐resistant) plasmid. The pBADGM/Myc‐HisA plasmid was modified from the pBAD/Myc‐HisA plasmid, differing only in its resistance gene^22^.

### Growth curve assays

A recently cultivated *E. coli* BL21 (DE3) strain carrying a pBAD/Myc‐HisA‐based vector was suspended to an OD_600_ of 0.03 and distributed in six replicate tubes: three tubes for the uninduced groups and three tubes for the arabinose‐induced groups. At 0 h, bacteria were stimulated with 2% l‐arabinose, and then incubated at 37°C with shaking. Cell growth was recorded hourly at OD_600_.

### Motility test

The MG1655 motility test was performed as previously described^25^. *E. coli* strains were grown overnight in LB broth and 2 μl of cultures was spotted onto soft LB plates containing 0.25% agar. Expression of the pBAD/Myc‐HisA vector was induced with 0.1% l‐arabinose. Swimming motility was observed after 6 h incubation at 37°C.

The twitching motility assays of *L. enzymogenes* OH11 were performed according to our earlier studies^38^, ^39^. Typically, sterile microscope slides were coated with a thin layer of 1 ml of 1/20 tryptic soy agar medium supplemented with 1.8% agar. The edge of the sterilized coverslip was placed in the bacterial solution and gently pressed onto the surface of the culture medium to form a narrow inoculation line. After 24 h of incubation, the edge of the bacterial culture on slide was examined under a microscope at ×640 magnification. According to our previous report^38^, the twitching motility of *L. enzymogenes* was characterized by the presence of a single mobile cell or a tiny cluster of mobile cells extending outward from the center of the colony. All experiments were repeated three times, using three replicates.

### RT‐PCR and quantitative real‐time PCR (qRT‐PCR)

RT‐PCR and qRT‐PCR were performed as previously described^34^. Briefly, *L. enzymogenes* strain OH11 and its derivatives were grown in LB medium with 25 μg/ml of Km overnight. 1% volume of cells was then transferred to LB medium and incubated at 28°C with shaking until the OD_600_ reached 1.0 in the exponential growth phase. Cells were then collected by centrifugation (12,000 rpm) at 4°C to extract total RNA using the Bacterial RNA Kit (no. R6950‐01; OMEGA) following the manufacturer's instructions. RNA integrity and concentration were assessed using a Thermo Scientific NanoDrop 2000. Next, RNA samples were used for complementary DNA (cDNA) synthesis using the PrimerScript™ RT reagent Kit with gDNA Eraser (no. RR047A; Takara). The primers used for the qRT‐PCR assays are listed in Table S8, and the 16S rRNA gene was used as an internal control as previously described^34^.

### Gene cotranscriptional assays

The RT‐PCR assays were based on an earlier study in our laboratory^38^. The RNA extraction method was the same as qRT‐PCR. Primers designed for RT‐PCR are listed in Table S9. The RT‐PCR assay was performed using the reverse‐transcribed cDNA as a template and genomic DNA as a positive control.

### Pull‐down assays

Plasmids pBAD/Myc‐HisA and pBADGM/Myc‐HisA of the bacterial two‐hybrid system were modified for pull‐down experiments. In brief, pBAD/Myc‐HisA containing Le1637‐Myc/Xac2247‐His and pBADGM/Myc‐HisA containing Le1636‐FLAG/Xac2246‐FLAG were cotransformed into *E. coli* BL21 (DE3) by electroporation. Transformed *E. coli* BL21 (DE3) were grown in LB (20 ml) until the OD_600_ reached 1.0. Cells were subsequently collected by centrifugation (12,000 rpm for 10 min at 4°C). Harvested cells were resuspended in 2 ml of 10 mM phosphate‐buffered saline (PBS, pH, 7.4), followed by sonication (Sonifier 250; Branson Digital Sonifier). After centrifugation (12,000 rpm for 10 min at 4°C), 1 ml of soluble protein was mixed with 30 μl of anti‐Myc/anti‐FLAG magnetic beads (Bimake) according to the manufacturer's instructions. After incubation overnight at 4°C, the beads were washed five times for 10 min each with 1 ml of 10 mM PBS buffer (pH 7.4) containing 1% Triton X‐100. Bead‐eluted proteins of Le1637‐Le1636 were identified by Western blots using the specific anti‐Myc (No. M20002L; Abmart) and anti‐FLAG (No. M20008S; Abmart) monoclonal antibodies, while Xac2247‐Xac2246 were identified by Western blots using specific anti‐FLAG (No. M20008S; Abmart) and anti‐His (No. M30111L; Abmart) monoclonal antibodies. Bound proteins were eluted with 45 μl of elution buffer (0.2 M glycine, pH 3.0), followed by 5 μl of neutralization buffer (1.5 M Tris, pH 10). The eluted protein pair of Le1637‐Le1636 was identified by Western blots using specific anti‐Myc (No. M20002L; Abmart) and anti‐FLAG (No. M20008S; Abmart) monoclonal antibodies, while the Xac2247‐Xac2246 pair was identified by Western blots using specific anti‐FLAG (No. M20008S; Abmart) and anti‐His (No. M30111L; Abmart) monoclonal antibodies.

### HSAF extraction and quantification

HSAF was extracted as previously described^34^. Briefly, 20 ml of the wild‐type strain or one of its variants was cultured with shaking in 1/10 tryptic soy broth at 28°C for 24 h. Cell‐free supernatants were obtained after centrifugation and mixed with an equal volume of ethyl acetate. After shaking for an hour, the ethyl acetate phase was recovered and evaporated to dryness. The HSAF‐containing residue was dissolved in 200 μl of methanol and passed through high‐performance liquid chromatography (HPLC). The peak areas on the HPLC chromatograms were normalized to the culture OD_600_ and used to calculate the relative amount of HSAF.

### Contact‐dependent killing assay

For fluorescent microscopy, the plasmid pYC12^22^ carrying the *mCherry* gene driven by a plasmid‐bearing constitutive promoter (*P*_tac_) was introduced into the *L. enzymogenes* strains, and plasmid pmCherry78 with a constitutively expressed *mCherry* gene was transformed into *E. cloacae* AA4^28^. The plasmid pMSC21 containing the constitutively expressed *GFP* gene was transferred into the competing strain *E. coli* BL21^37^. Similarly, plasmid pBBR1‐MCS5 with a constitutively expressed *GFP* gene was transformed into *P. fluorescens* 2P24^40^ and *P. protegens* Pf‐5^41^, and plasmid pGFP78 with constitutively expressed *GFP* gene was transformed into *E. cloacae* AA4^28^. After overnight incubation in LB medium at 28°C in an orbital shaker (200 rpm), all bacterial cells were collected by centrifugation (6000 rpm for 3 min at room temperature) and suspended in fresh LB to a final OD_600_ of 1.0. A volume of 750 μl of the resulting *L. enzymogenes* strain cell suspension was mixed with other strains (*E. coli* BL21, *P. fluorescens* 2P24, *P. protegens* Pf‐5, and *E. cloacae* AA4) at a ratio of 1:1. Then, a volume of 5 μl of the mixture culture was spot‐inoculated on LA dishes, followed by incubation at 28°C for 24 h. A 0.22‐μM filter membrane was used to separate the growth of OH11 and AA4. A stereoscopic fluorescence microscope (Nikon SMZ25; Nikon) was used to observe the fluorescence signal under excitation at 488 and 561 nm for GFP and mCherry, respectively. Subsequently, each coinoculated colony was picked and resuspended in fresh LB (1 ml). Suspended bacterial cells (150 μl) were grown in antibiotic‐free LA plates at 28°C for 3 days, and then competing colonies were counted by GFP fluorescence detected using a stereo fluorometer (Nikon SMZ25; Nikon). Afterward, the number of c.f.u. of recovered prey cells was determined and counted. All experiments were carried out three times and each treatment was repeated three times. Comparisons of mean values were carried out using Student's *t* test (*α* = 0.05) implemented in the SPSS 14.0 package (SPSS).

## AUTHOR CONTRIBUTIONS

**Xi Shen**: Conceptualization (lead); data curation (equal); formal analysis (equal); methodology (equal); project administration (equal); resources (lead); software (equal); supervision (equal); writing—original draft (equal); writing—review and editing (equal). **Zixiang Yang**: Data curation (equal); resources (equal). **Zihan Li**: Conceptualization (equal); data curation (equal); resources (equal); software (equal). **Dan Xiong**: Conceptualization (equal); Data curation (equal); resources (equal). **Jinxing Liao**: Conceptualization (equal); resources (equal). **Weimei He**: Conceptualization (equal); resources (equal). **Danyu Shen**: Data curation (equal); software (equal). **Xiaolong Shao**: Funding acquisition (equal); validation (equal). **Ben Niu**: Formal analysis (equal); resources (supporting). **Yongxing He**: Formal analysis (equal); validation (supporting). **Yong‐Gui Gao**: Funding acquisition (supporting); validation (equal). **Guoliang Qian**: Data curation (equal); formal analysis (equal); funding acquisition (lead); project administration (lead); writing—original draft (lead); writing—review and editing (lead).

## ETHICS STATEMENT

This study did not involve human subjects and animals.

## CONFLICT OF INTERESTS

The authors declare no conflict of interests.

## Supporting information

Supporting information.

## Data Availability

The raw DNA‐seq data that support the findings of this study are openly available in the NCBI GeneBank database under accession number OQ972018 for Le1637, OQ972017 for Le1636, OQ972016 for Le3432 and OQ972015 for Le4802.
