# Supplementary material for: Identification of atypical T4SS effector proteins mediating bacterial defense
Source: mLife. 2023 Sep 24;2(3):295–307. doi: 10.1002/mlf2.12084 (PMC10989847; doi:10.1002/mlf2.12084)
Supplement: Supplementary file 1 — Supporting information. [file MLF2-2-295-s001.docx]

**Supplemental materials**

**Identification of atypical T4SS effector proteins mediating bacterial defense**

Xi Shen^a^, Zixiang Yang^a^, Zihan Li^a^, Dan Xiong^a^, Jinxing Liao^a^, Weimei He^a^, Danyu Shen^a^, Xiaolong Shao^a^, Ben Niu^b^, Yongxing He^c^, Yong-Gui Gao^d^, Guoliang Qian^a^*

**Contains 9 supplemental tables and 7 supplemental figures.**

**Table S1. Homology comparison with Type IV secretion system effectors in selected species that carry an X-T4SS**

| **Protein name** | **Length**  **（aa）** | **Description** | **Accession number** | **Protein name** | **Length（aa）** | **Description** | **Accession number** | **Percentage of Identity（%）** |
| --- | --- | --- | --- | --- | --- | --- | --- | --- |
| *Lysobacter enzymogenes* OH11 | | | | | | | | |
| T4SS Effector | | | | Homologous Protein | | | | |
| Le4230 | 414 | hypothetical protein | None | Le5014 | 609 | mannosyl-glycoprotein endo-beta-N-acetylglucosamidase | None | 52.30 |
| Le0908 | 480 | hypothetical protein | None | Le3870 | 449 | conserved hypothetical protein | None | 41.77 |
| Le3432 | 402 | phospholipase A1 | OQ972016 | Le1637 | 321 | Extracellular phospholipase A1 | OQ972018 | 44.69 |
| Le1288 | 540 | lipase | None | Le1653 | 397 | lipase | None | 41.34 |
| *Xanthomonas crtri* 306 | | | | | | | | |
| T4SS Effector | | | | Homologous Protein | | | | |
| XAC3266 | 861 | hypothetical protein | AAM38110.1 | XAC2506 | 1309 | hypothetical protein | AAM37357.1 | 66.11 |
|  |  |  |  | XAC2636 | 661 | hypothetical protein | AAM37483.1 | 44.19 |
|  |  |  |  | XAC2247 | 854 | conserved hypothetical protein | AAM37100.1 | 41.15 |
|  |  |  |  | XAC2861 | 417 | hypothetical protein | AAM37706.1 | 41.01 |
|  |  |  |  | XAC2860 | 636 | hypothetical protein | AAM37705.1 | 52.38 |
| *Stenotrophomonas maltophilia* K279a | | | | | | | | |
| T4SS Effector | | | | Homologous Protein | | | | |
| Smlt2992 | 334 | Putative bacteriophage protein | CAQ46442.1 | Smlt0315 | 211 | Putative phage lytic enzyme | CAQ43918.1 | 32.60 |
| *Lysobacter antibioticus* 76 | | | | | | | | |
| T4SS Effector | | | | Homologous Protein | | | | |
| LA76x_2414 | 453 | putative peptidoglycan binding domain protein | ALN80544.1 | LA76x_1288 | 212 | chitinase class I family protein | ALN79446.1 | 24.50 |
| LA76x_2419 | 425 | putative peptidoglycan binding domain protein | ALN80549.1 | LA76x_0978 | 1295 | putative peptidoglycan binding domain protein | ALN79139.1 | 21.10 |
| LA76x_2436 | 449 | putative peptidoglycan binding domain protein | ALN80566.1 | LA76x_0978 | 1295 | putative peptidoglycan binding domain protein | ALN79139.1 | 21.40 |
| LA76x_3167 | 682 | zeta toxin family protein | ALN81295.1 | LA76x_5016 | 1257 | putative peptidoglycan binding domain protein | ALN83118.1 | 23.40 |
| LA76x_0696 | 455 | lipase family protein | ALN78857.1 | LA76x_4483 | 450 | lipase family protein | ALN82591.1 | 54.30 |
| LA76x_4983 | 451 | lipase family protein | ALN83085.1 | LA76x_4483 | 450 | lipase family protein | ALN82591.1 | 58.30 |
| LA76x_3666 | 403 | hypothetical protein | ALN81788.1 | LA76x_4848 | 504 | extracellular phospholipase A1 domain protein | ALN82951.1 | 42.50 |
| LA76x_4493 | 603 | hypothetical protein | ALN82601.1 | LA76x_1464 | 609 | hypothetical protein | ALN79620.1 | 39.30 |
| LA76x_4497 | 599 | hypothetical protein | ALN82605.1 | LA76x_1464 | 609 | hypothetical protein | ALN79620.1 | 36.60 |
| LA76x_2624 | 584 | hypothetical protein | ALN80754.1 | LA76x_1464 | 609 | hypothetical protein | ALN79620.1 | 36.80 |
| LA76x_4281 | 486 | hypothetical protein | ALN82392.1 | LA76x_1464 | 609 | hypothetical protein | ALN79620.1 | 43.40 |
| LA76x_3256 | 459 | hypothetical protein | ALN81383.1 | LA76x_1464 | 609 | hypothetical protein | ALN79620.1 | 40.50 |
| LA76x_2831 | 355 | hypothetical protein | ALN80961.1 | LA76x_4848 | 504 | extracellular phospholipase A1 domain protein | ALN82951.1 | 35.30 |
| *Lysobacter enzymegenes* C3 | | | | | | | | |
| T4SS Effector | | | | Homologous Protein | | | | |
| GLE_2806 | 437 | putative peptidoglycan binding domain protein | ALN58154.1 | GLE_0196 | 1205 | large Ala/Gln-rich protein | ALN55555.1 | 21.00 |
| GLE_4488 | 760 | putative peptidoglycan binding domain protein | ALN59829.1 | GLE_0196 | 1205 | large Ala/Gln-rich protein | ALN55555.1 | 28.50 |
| GLE_2788 | 448 | putative peptidoglycan binding domain protein | ALN58136.1 | GLE_0196 | 1205 | large Ala/Gln-rich protein | ALN55555.1 | 20.60 |
| GLE_4797 | 540 | hypothetical protein | ALN60138.1 | GLE_5184 | 402 | hypothetical protein | ALN60525.1 | 43.30 |
| GLE_0046 | 451 | hypothetical protein | ALN55405.1 | GLE_5184 | 402 | hypothetical protein | ALN60525.1 | 41.00 |
| GLE_0598 | 443 | hypothetical protein | ALN55956.1 | GLE_5184 | 402 | hypothetical protein | ALN60525.1 | 55.10 |
| GLE_1521 | 402 | phospholipase A1 | ALN56878.1 | GLE_5168 | 321 | Extracellular phospholipase A1 | ALN60509.1 | 46.50 |
| *Luteibacter rhizovicinus* DSM16549 | | | | | | | | |
| T4SS Effector | | | | Homologous Protein | | | | |
| BJI69_13815 | 424 | hypothetical protein | APG04863.1 | BJI69_14270 | 177 | chitinase | APG04942.1 | 23.10 |
| *Neisseria mucosa* C102 | | | | | | | | |
| T4SS Effector | | | | Homologous Protein | | | | |
| HMPREF0604_RS05620 | 460 | calcium-binding protein | WP_231288098.1 | HMPREF0604_RS03275 | 141 | hypothetical protein | WP_050787144.1 | 23.30 |
| HMPREF0604_RS03260 | 441 | hypothetical protein | WP_003746729.1 | HMPREF0604_RS05925 | 440 | DUF2974 domain-containing protein | WP_003747789.1 | 57.60 |
| HMPREF0604_RS05990 | 316 | hypothetical protein | WP_003747822.1 | HMPREF0604_RS05925 | 440 | DUF2974 domain-containing protein | WP_003747789.1 | 40.60 |

**Table S2. Prediction of type II TA system in *Xanthomonas citri* 306 by TADB2**

|  | **Gene ID** | **T/A** | **Functional annotation** |
| --- | --- | --- | --- |
| Pair1 | XAC0080 | Antitoxin | conserved hypothetical protein |
|  | XAC0081 | Toxin | conserved hypothetical protein |
| Pair2 | XAC0188 | Antitoxin | transcriptional regulator |
|  | XAC0187 | Toxin | HipA protein |
| Pair3 | XAC0267 | Antitoxin | conserved hypothetical protein |
|  | XAC0268 | Toxin | conserved hypothetical protein |
| Pair4 | XAC0508 | Antitoxin | transcriptional regulator lysR family |
|  | XAC0507 | Toxin | 2-acylglycerophosphoethanolamine acyltransferase |
| Pair5 | XAC0769 | Antitoxin | conserved hypothetical protein |
|  | XAC0768 | Toxin | conserved hypothetical protein |
| Pair6 | XAC1140 | Antitoxin | conserved hypothetical protein |
|  | XAC1141 | Toxin | conserved hypothetical protein |
| Pair7 | XAC1195 | Antitoxin | hypothetical protein |
|  | XAC1194 | Toxin | death-on-curing protein |
| Pair8 | XAC1493 | Antitoxin | transcriptional regulator |
|  | XAC1494 | Toxin | phage-related protein |
| Pair9 | XAC1499 | Antitoxin | transcriptional regulator |
|  | XAC1501 | Toxin | hypothetical protein |
| Pair10 | XAC1883 | Antitoxin | conserved hypothetical protein |
|  | XAC1884 | Toxin | conserved hypothetical protein |
| Pair11 | XAC2188 | Antitoxin | cell growth regulatory protein |
|  | XAC2187 | Toxin | conserved hypothetical protein |
| Pair12 | XAC2429 | Antitoxin | conserved hypothetical protein |
|  | XAC2428 | Toxin | conserved hypothetical protein |
| Pair13 | XAC3974 | Antitoxin | two-component system regulatory protein |
|  | XAC3973 | Toxin | cell division inhibitor |
| Pair14 | XAC4312 | Antitoxin | conserved hypothetical protein |
|  | XAC4313 | Toxin | conserved hypothetical protein |
| Pair15 | XAC4314 | Antitoxin | plasmid stability protein |
|  | XAC4313 | Toxin | conserved hypothetical protein |
| Pair16 | XAC4314 | Antitoxin | plasmid stability protein |
|  | XAC4315 | Toxin | plasmid stability protein |

**Table S3. Prediction of type II TA system in *Lysobacter enzymogenes* C3 by TADB2**

|  | **Gene ID** | **T/A** | **Functional annotation** |
| --- | --- | --- | --- |
| Pair1 | GLE_2121 | Antitoxin | Aha1 domain protein |
|  | GLE_2122 | Toxin | transcriptional regulator, ArsR family |
| Pair2 | GLE_2213 | Antitoxin | hypothetical protein |
|  | GLE_2212 | Toxin | RES domain superfamily |
| Pair3 | GLE_2296 | Antitoxin | transcriptional regulator, AbrB family |
|  | GLE_2297 | Toxin | hypothetical protein |
| Pair4 | GLE_4010 | Antitoxin | arsenical resistance operon repressor |
|  | GLE_4011 | Toxin | acetyltransferase, GNAT family |
| Pair5 | GLE_4108 | Antitoxin | hypothetical protein |
|  | GLE_4107 | Toxin | RelE/ParE family protein |
| Pair6 | GLE_4144 | Antitoxin | hypothetical protein |
|  | GLE_4143 | Toxin | lipoprotein, LppC family |
| Pair7 | GLE_4266 | Antitoxin | Clp regulator protein |
|  | GLE_4267 | Toxin | acetyltransferase (GNAT) family |
| Pair8 | GLE_4398 | Antitoxin | transcriptional regulator, ArsR family |
|  | GLE_4397 | Toxin | glutathione S-transferase protein |
| Pair9 | GLE_4398 | Antitoxin | transcriptional regulator, ArsR family |
|  | GLE_4399 | Toxin | hypothetical protein |
| Pair10 | GLE_4886 | Antitoxin | arsenical resistance operon repressor |
|  | GLE_4887 | Toxin | Aha1 domain superfamily |

**Table S4. Prediction of type II TA system in *Luteibacter rhizovicinus* DSM16549 by TADB2**

|  | **Gene ID** | **T/A** | **Functional annotation** |
| --- | --- | --- | --- |
| Pair1 | BJI69_06160 | Antitoxin | hypothetical protein |
|  | BJI69_06155 | Toxin | hypothetical protein |
| Pair2 | BJI69_06440 | Antitoxin | prevent-host-death protein |
|  | BJI69_06445 | Toxin | plasmid stabilization protein |
| Pair3 | BJI69_06745 | Antitoxin | AsnC family transcriptional regulator |
|  | BJI69_06740 | Toxin | 1-acyl-sn-glycerol-3-phosphate acyltransferase |
| Pair4 | BJI69_06770 | Antitoxin | DUF2384 domain-containing protein |
|  | BJI69_06765 | Toxin | hypothetical protein |
| Pair5 | BJI69_07290 | Antitoxin | LysR family transcriptional regulator |
|  | BJI69_07285 | Toxin | MFS transporter |
| Pair6 | BJI69_09470 | Antitoxin | transcriptional regulator |
|  | BJI69_09465 | Toxin | toxin HigB-2 |
| Pair7 | BJI69_10715 | Antitoxin | hypothetical protein |
|  | BJI69_10710 | Toxin | hypothetical protein |
| Pair8 | BJI69_11510 | Antitoxin | histidine kinase |
|  | BJI69_11515 | Toxin | glycosyl transferase family 2 |
| Pair9 | BJI69_12545 | Antitoxin | addiction module antidote protein, HigA family |
|  | BJI69_12540 | Toxin | hypothetical protein |
| Pair10 | BJI69_17130 | Antitoxin | hypothetical protein |
|  | BJI69_17125 | Toxin | hypothetical protein |
| Pair11 | BJI69_18065 | Antitoxin | transcriptional regulator |
|  | BJI69_18060 | Toxin | ATPase |
| Pair12 | BJI69_18725 | Antitoxin | hypothetical protein |
|  | BJI69_18720 | Toxin | toxin HipA |
| Pair13 | BJI69_20835 | Antitoxin | hypothetical protein |
|  | BJI69_20830 | Toxin | MFS transporter |
| Pair14 | BJI69_20865 | Antitoxin | putative addiction module antidote protein |
|  | BJI69_20870 | Toxin | addiction module antitoxin RelB |

**Table S5. Prediction of type II TA system in *Stenotrophomonas maltophilia* K279a by TADB2**

|  | **Gene ID** | **T/A** | **Functional annotation** |
| --- | --- | --- | --- |
| Pair1 | Smlt0067 | Antitoxin | uncharacterized protein |
|  | Smlt0068 | Toxin | uncharacterized protein |
| Pair2 | Smlt0135 | Antitoxin | putative HTH-type transcriptional regulator HipB |
|  | Smlt0134 | Toxin | putative survival protein HipA |
| Pair3 | Smlt0334 | Antitoxin | putative phage-related protein |
|  | Smlt0335 | Toxin | putative HTH family transcriptional regulator |
| Pair4 | Smlt0807 | Antitoxin | putative ISPsy9 like transposase |
|  | Smlt0806 | Toxin | putative transmembrane MFS family protein |
| Pair5 | Smlt1305 | Antitoxin | putative MerR family transcriptional regulator |
|  | Smlt1306 | Toxin | putative MFS family transmembrane transporter |
| Pair6 | Smlt1334 | Antitoxin | uncharacterized protein |
|  | Smlt1335 | Toxin | uncharacterized protein |
| Pair7 | Smlt1817 | Antitoxin | putative ArsR family transcriptional regulator |
|  | Smlt1818 | Toxin | putative Hsp90 family heat shock chaperone protein |
| Pair8 | Smlt1898 | Antitoxin | putative HTH transcriptional regulator |
|  | Smlt1897 | Toxin | uncharacterized protein |
| Pair9 | Smlt2090 | Antitoxin | putative ArsR family regulatory protein |
|  | Smlt2089 | Toxin | uncharacterized protein |
| Pair10 | Smlt2090 | Antitoxin | putative ArsR family regulatory protein |
|  | Smlt2091 | Toxin | putative Hsp90 ATPase like protein |
| Pair11 | Smlt2118 | Antitoxin | sulfurtransferase |
|  | Smlt2117 | Toxin | putative acetyltransferase |
| Pair12 | Smlt2243 | Antitoxin | putative SpoVT/AbrB domain transcriptional regulatory protein |
|  | Smlt2244 | Toxin | uncharacterized protein |
| Pair13 | Smlt2677 | Antitoxin | putative TetR-family regulatory protein |
|  | Smlt2678 | Toxin | putative transmembrane protein |
| Pair14 | Smlt3607 | Antitoxin | uncharacterized protein |
|  | Smlt3606 | Toxin | putative plasmid stabilisation system protein |
| Pair15 | Smlt4255 | Antitoxin | putative DNA-binding protein |
|  | Smlt4256 | Toxin | uncharacterized protein |

**Table S6. Distribution of Le1637 in different bacteria**

| **Organisms** | **Gene ID** | **Per. Ident（%）** |
| --- | --- | --- |
| *Lysobacter silvisoli* | WP_115858642.1 | 48.4 |
| *Lysobacter capsici* AZ78 | WP_046658641.1 | 87.7 |
| *Lysobacter antibioticus* | WP_152566317.1 | 39.7 |
| *Lysobacter gummosus* | WP_057941167.1 | 88.9 |
| *Lysobacter terrestris* | WP_187712546.1 | 45.5 |
| *Lysobacter alkalisoli* | WP_141625035.1 | 50.6 |
| *Luteimonas arsenica* | WP_132999387.1 | 72.9 |
| *Luteimonas padinae* | WP_189494213.1 | 72 |
| *Luteimonas gilva* | WP_137266651.1 | 72.2 |
| *Luteimonas fraxinea* | WP_232148255.1 | 71.7 |
| *Luteimonas aquatica* | WP_242112779.1 | 69.2 |
| *Luteimonas terrae* | WP_133393972.1 | 70.1 |
| *Luteimonas salinisoli* | WP_180677957.1 | 72.3 |
| *Luteimonas panaciterrae* | WP_226467462.1 | 66.9 |
| *Luteimonas cucumeris* | WP_144900538.1 | 72.3 |
| *Pseudoxanthomonas sacheonensis* | WP_163838796.1 | 44.8 |
| *Pseudoxanthomonas yeongjuensis* | WP_162313741.1 | 46.1 |
| *Pseudoxanthomonas suwonensis* | PZO64524.1 | 67.6 |
| *Coralloluteibacterium stylophorae* | WP_211925381.1 | 65.4 |
| *Dyella acidiphila* | WP_192556287.1 | 44.5 |
| *Yersinia frederiksenii* | WP_050123000.1 | 60.2 |
| *Yersinia alsatica* | WP_145599360.1 | 60.2 |
| *Yersinia kristensenii* | WP_087794980.1 | 60.4 |
| *Yersinia enterocolitica* | WP_050138547.1 | 59.9 |
| *Xanthomonas surreyensis* | WP_191825694.1 | 45.2 |
| *Xanthomonas bonasiae* | WP_206259410.1 | 45.7 |
| *Xanthomonas arboricola* | WP_104563365.1 | 44.7 |
| *Xanthomonas campestris* | MBN8211624.1 | 45.4 |
| *Xanthomonas vesicatoria* | WP_228862025.1 | 44.6 |
| *Xanthomonas translucens* | WP_053842116.1 | 45.5 |
| *Xanthomonas cucurbitae* | WP_159407297.1 | 43.3 |
| *Photorhabdus temperata* | WP_021324747.1 | 51.3 |
| *Photorhabdus khanii* | WP_132353084.1 | 52.5 |
| *Photorhabdus stackebrandtii* | WP_166290912.1 | 52.5 |
| *Photorhabdus tasmaniensis* | WP_133815353.1 | 53.1 |
| *Serratia liquefaciens* | WP_262241795.1 | 60.1 |
| *Serratia proteamaculans* | WP_153859336.1 | 62 |
| *Serratia plymuthica* | WP_062868314.1 | 60.1 |
| *Xenorhabdus bovienii* | WP_143827684.1 | 52.6 |
| *Xenorhabdus doucetiae* | WP_045971803.1 | 55.3 |
| *Xenorhabdus nematophila* | WP_041979514.1 | 49.5 |

**Table S7. Distribution of XAC2247 in different bacteria**

| **Organisms** | **Gene ID** | **Per. Ident（%）** |
| --- | --- | --- |
| *Xanthomonas citri* | AAM37100.1 | 100 |
| *Xanthomonas axonopodis* | WP_139370408.1 | 62.8 |
| *Xanthomonas phaseoli* | WP_244665539.1 | 59 |
| *Xanthomonas campestris* | WP_227971697.1 | 56.9 |
| *Xanthomonas arboricola* | WP_245230330.1 | 56.3 |
| *Xanthomonas hydrangeae* | CAD7734565.1 | 56.5 |
| *Xanthomonas hortorum* | WP_023904495.1 | 56.1 |
| *Xanthomonas vasicola* | WP_244220715.1 | 57.7 |
| *Xanthomonas cassavae* | WP_152527265.1 | 51.9 |
| *Xanthomonas melonis* | WP_228329391.1 | 56.9 |
| *Xanthomonas cucurbitae* | WP_159407923.1 | 53.8 |
| *Xanthomonas codiaei* | WP_228964935.1 | 55.6 |
| *Xanthomonas prunicola* | WP_101362505.1 | 53.9 |
| *Stenotrophomonas indicatrix* | WP_143062981.1 | 59.5 |
| *Stenotrophomonas maltophilia* | WP_262223474.1 | 46.6 |
| *Pantoea ananatis* | WP_264400739.1 | 46.4 |
| *Acidovorax cattleyae* | WP_092834578.1 | 50.5 |
| *Acidovorax citrulli* | WP_011793715.1 | 50.2 |
| *Acidovorax valerianellae* | WP_217640130.1 | 51.2 |
| *Acidovorax avenae* | WP_128098935.1 | 50.4 |

**Table S8. Strains and plasmids used in this study**

| **Strains and plasmids** | **Characteristics^a^** | **Source** |
| --- | --- | --- |
| *Lysobacter enzymogenes* | | |
| OH11 | Wild type, Km^R^ | ^1^ |
| Δ*Le1637* | In-frame deletion of *Le1637*, Km^R^ | This study |
| Δ*Le3432* | In-frame deletion of *Le3432*, Km^R^ | This study |
| Δ*Le4802* | In-frame deletion of *Le4802*, Km^R^ | This study |
| Δ*Le1637^S186A^* | In-frame *Le1637S186* mutation, Km^R^ | This study |
| Δ*Le1637^E243A^* | In-frame *Le1637E243* mutation, Km^R^ | This study |
| Δ*Le1637*-Δ*Le1636* | In-frame deletion of *Le1637* and *Le1636*, Km^R^ | This study |
| OH11 (mCherry) | OH11 harboring plasmid pYC12-mCherry, Gm^R^, Km^R^ | ^2^ |
| Δ*Le1637* (mCherry) | Δ*Le1637* harboring plasmid pYC12-mCherry, Gm^R^, Km^R^ | This study |
| Δ*Le3432* (mCherry) | Δ*Le3432* harboring plasmid pYC12-mCherry, Gm^R^, Km^R^ | This study |
| Δ*Le4802* (mCherry) | Δ*Le4802* harboring plasmid pYC12-mCherry, Gm^R^, Km^R^ | This study |
| OH11 (Le1637-GFP) | OH11 harboring plasmid pBBR1-GFP-Le1637, Gm^R^, Km^R^ | This study |
| *Escherichia coli* | | |
| DH5α | Host strain for molecular cloning | ^3^ |
| BL21 (DE3) | Host strain for molecular cloning | ^4^ |
| MG1655 | Wild-type, host strain for protein expression in swimming motility assay | Lab collection |
| BL21 (GFP) | BL21 harboring pSMC21-GFP vector, Km^R^ | Lab collection |
| *Pseudomonas fluorescence* | | |
| 2P24 (GFP) | 2P24 harboring pBBR1-MCS5-GFP vector, Km^R^, Gm^R^ | This study |
| *Pseudomonas protegens* | | |
| Pf-5 (GFP) | Pf-5 harboring pBBR1-MCS5-GFP vector, Amp^R^, Gm^R^ | This study |
| *Enterobacter cloacae* | | |
| AA4 (GFP) | GFP labeled strain AA4, Tet^R^ | ^5^ |
| Plasmids | | |
| pEX18GM | Suicide vector with a *sacB* gene, Gm^R^ | ^6^ |
| pEX18GM-*Le1637* | pEX18GM with two flanking fragments of *Le1637*, Gm^R^ | This study |
| pEX18GM-*Le3432* | pEX18GM with two flanking fragments of *Le3432*, Gm^R^ | This study |
| pEX18GM-*Le4802* | pEX18GM with two flanking fragments of *Le4802*, Gm^R^ | This study |
| pEX18GM-*Le1636* | pEX18GM with two flanking fragments of *Le1636*, Gm^R^ | This study |
| pBBR1-MCS5 | Broad-host-range vector with a P*_lac_* promoter, Gm^R^ | ^7^ |
| pBBR1-GFP | pBBR1-MCS5 containing the coding region of GFP, Gm^R^ | ^2^ |
| pBBR1-GFP-FLAG | pBBR1-MCS5 containing the coding region of GFP with C-terminal FLAG tag and its native promoter, Gm^R^ | This study |
| pBBR1-GFP-Le1637 | pBBR1-MCS5 containing the coding region of GFP, Le1637 and its native promoter, Gm^R^ | This study |
| pBAD/Myc-His A | Vector for arabinose-inducible gene expression, Amp^R^ | ^7^ |
| pBAD-AvrRXO1 | pBAD containing the coding region of AvrRXO1, Amp^R^ | ^8^ |
| pBAD-XAC2506 | pBAD containing the coding region of XAC2506, Amp^R^ | This study |
| pBAD-XAC2247 | pBAD containing the coding region of XAC2247, Amp^R^ | This study |
| pBAD-XAC2861 | pBAD containing the coding region of XAC2861, Amp^R^ | This study |
| pBAD-XAC2860 | pBAD containing the coding region of XAC2860, Amp^R^ | This study |
| pBAD-XAC2247-His | pBAD containing the coding region of XAC2247 with C-terminal His tag and its native promoter, Amp^R^ | This study |
| pBAD-Le3870 | pBAD containing the coding region of Le3870, Amp^R^ | This study |
| pBAD-Le1653 | pBAD containing the coding region of Le1653, Amp^R^ | This study |
| pBAD-Le5014 | pBAD containing the coding region of Le5014, Amp^R^ | This study |
| pBAD-Le1637 | pBAD containing the coding region of Le1637, Amp^R^ | This study |
| pBAD-Le0849 | pBAD containing the coding region of Le0849, Amp^R^ | This study |
| pBAD-Le1374 | pBAD containing the coding region of Le1374, Amp^R^ | This study |
| pBAD-Le4706 | pBAD containing the coding region of Le4706, Amp^R^ | This study |
| pBAD-Le4802 | pBAD containing the coding region of Le4802, Amp^R^ | This study |
| pBAD-Le4886 | pBAD containing the coding region of Le4886, Amp^R^ | This study |
| pBAD-Le1637^S186A^ | pBAD containing the coding region of Le1637 with a S186 substitution by A186, Amp^R^ | This study |
| pBAD-Le1637^E243A^ | pBAD containing the coding region of Le1637 with a E243 substitution by A243, Amp^R^ | This study |
| pBAD-Le1637-Myc | pBAD containing the coding region of Le1637 with C-terminal Myc tag and its native promoter, Amp^R^ | This study |
| pBAD-GFP-Myc | pBAD containing the coding region of GFP with C-terminal Myc tag and its native promoter, Amp^R^ | This study |
| pBADGM | Vector for arabinose-inducible gene expression, Gm^R^ | ^2^ |
| pBADGM-XAC2246-FLAG | pBADGM containing the coding region of XAC2246 with C-terminal FLAG tag and its native promoter, Gm^R^ | This study |
| pBADGM-XAC2248-FLAG | pBADGM containing the coding region of XAC2248 with C-terminal FLAG tag and its native promoter, Gm^R^ | This study |
| pBADGM-Le1636-FLAG | pBADGM containing the coding region of Le1636 with C-terminal FLAG tag and its native promoter, Gm^R^ | This study |
| pBADGM-Le1638-FLAG | pBADGM containing the coding region of Le1638 with C-terminal FLAG tag and its native promoter, Gm^R^ | This study |
| pYC12-mCherry | pYC12 containing the coding region of mCherry, Gm^R^ | ^9^ |

**Table S9. Primers used in this study**

| **Primer** | **Sequence (5'-3')^a^** | **Purpose** |
| --- | --- | --- |
| In-frame deletion | | |
| *Le1637*-F1 | CGGGATCCTACTGCGGATCGTTGGCGAT (*BamH*I) | To amplify a 522-bp fragment upstream of *Le1637* |
| *Le1637*-R1 | CCCAAGCTTCGATCGAGATGGCCAAGCGC (*Hind*III) |  |
| *Le1637*-F2 | CCCAAGCTTATGGCCTGCGAATCCTGGCC (*Hind*III) | To amplify a 574-bp fragment upstream of *Le1637* |
| *Le1637*-R2 | GCTCTAGATGCGGGTGGCGAAGGTCTTG (*Xba*I) |  |
| *Le3432*-F1 | CGGGATCCAAATCCAGTTCCGCCGCTTG (*BamH*I) | To amplify a 368-bp fragment upstream of *Le3432* |
| *Le3432*-R1 | CCCAAGCTTGGCGGTCGGTTCGGTCTGCT (*Hind*III) |  |
| *Le3432*-F2 | CCCAAGCTTTGAGCACGCCGCAGAACACC (*Hind*III) | To amplify a 243-bp fragment upstream of *Le3432* |
| *Le3432*-R2 | GCTCTAGAGTCTTGGTGGGAGGGGCTTC (*Xba*I) |  |
| *Le4802*-F1 | CGGGATCCATCCACCTGCTCGGCATCGC (*BamH*I) | To amplify a 356-bp fragment upstream of *Le4802* |
| *Le4802*-R1 | CCCAAGCTTGCTGGCCCTCGTCGTTCCAG (*Hind*III) |  |
| *Le4802*-F2 | CCCAAGCTTAGGAATACAACGCGCTGATC (*Hind*III) | To amplify a 225-bp fragment upstream of *Le4802* |
| *Le4802*-R2 | GCTCTAGAAAACGCTTCGCGGCAACACG (*Xba*I) |  |
| *Le1636*-F1 | ATGACCATGATTACGAATTCGTCGATGCGGGAAAGATGCG | To amplify a 300-bp fragment upstream of *Le1636* |
| *Le1636*-R1 | ATCGTTGCGTCGAGCCTGTTACGATGTGGCGGTGGAGGAC |  |
| *Le1636*-F2 | GTCCTCCACCGCCACATCGTAACAGGCTCGACGCAACGAT | To amplify a 174-bp fragment upstream of *Le1636* |
| *Le1636*-R2 | CGACGGCCAGTGCCAAGCTTTCGCCGACAAGGCCACCCGT |  |
| *Le1637^S186A^*-overlap-R | CAGGCCGCCGCCGAGCGCATGGCCGGTGATCAC | To amplify a 963-bp fragment containing coding region of Le1637 with a S186 substitution by A186 |
| *Le1637^S186A^*- overlap-F | GTGATCACCGGCCATGCGCTCGGCGGCGGCCTG |  |
| *Le1637^E243A^*-overlap-R | CGCCGCGGTCAGGATCGCGCCGCTGACGTTGTA | To amplify a 963-bp fragment containing coding region of Le1637 with a E243 substitution by A243 |
| *Le1637^E243A^*- overlap-F | TACAACGTCAGCGGCGCGATCCTGACCGCGGCG |  |
| Cytoplasmic expression in *Escherichia coli* | | |
| pBAD-XAC2506-F | ACAGGAGGAATTAACCATGGATTTGGAGATGCTGGCAGAAAA | To amplify a 3927-bp fragment containing coding region of XAC2506 |
| pBAD-XAC2506-R | TTTTGTTCGGGCCCAAGCTTACCGCCGCCTCCACCACCAC |  |
| pBAD-XAC2247-F | ACAGGAGGAATTAACCATGGATATGCCGGCCAACGATGCCAT | To amplify a 2562-bp fragment containing coding region of XAC2247 |
| pBAD-XAC2247-R | TTTTGTTCGGGCCCAAGCTTTCGATCCAAGGTTTCTTGCT |  |
| pBAD-XAC2860-F | ACAGGAGGAATTAACCATGGATTTGCAGCCGCAGTGGCGTGA | To amplify a 1908-bp fragment containing coding region of XAC2860 |
| pBAD-XAC2860-R | TTTTGTTCGGGCCCAAGCTTGTGCCTGGCGCGTACTCCCT |  |
| pBAD-XAC2861-F | ACAGGAGGAATTAACCATGGATTTGATCGACAAGGATGTATC | To amplify a 1251-bp fragment containing coding region of XAC2861 |
| pBAD-XAC2861-R | TTTTGTTCGGGCCCAAGCTTCGGATCCTGCGGAGCGGGCG |  |
| pBAD-Le3870-F | ACAGGAGGAATTAACCATGGATGTGTCCAATCTTGACGATCT | To amplify a 1371-bp fragment containing coding region of Le3870 |
| pBAD-Le3870-R | TTTTGTTCGGGCCCAAGCTTAATCGAGCGGCCACGATCGT |  |
| pBAD-Le1653-F | ACAGGAGGAATTAACCATGGATATGAGCCATACCAGCCCGCA | To amplify a 1191-bp fragment containing coding region of Le1653 |
| pBAD-Le1653-R | TTTTGTTCGGGCCCAAGCTTGGCCATGCGCGGGCCGCGCG |  |
| pBAD-Le5014-F | ACAGGAGGAATTAACCATGGATATGGCAGGGACACGCAGGCA | To amplify a 1827-bp fragment containing coding region of Le5014 |
| pBAD-Le5014-R | TTTTGTTCGGGCCCAAGCTTCTGCCTTGGAGTTCCCCGAT |  |
| pBAD-Le1637-F | ACAGGAGGAATTAACCATGGATATGAACGTGCAGCTGCAACC | To amplify a 963-bp fragment containing coding region of Le1637 |
| pBAD-Le1637-R | TTTTGTTCGGGCCCAAGCTTCTGCCAGGGCGTCTGCTGCT |  |
| pBAD-Le1637^S186A^-overlap-R | CAGGCCGCCGCCGAGCGCATGGCCGGTGATCAC | To amplify a 963-bp fragment containing coding region of Le1637 with a S186 substitution by A186 |
| pBAD-Le1637^S186A^- overlap-F | GTGATCACCGGCCATGCGCTCGGCGGCGGCCTG |  |
| pBAD-Le1637^E243A^-overlap-R | CGCCGCGGTCAGGATCGCGCCGCTGACGTTGTA | To amplify a 963-bp fragment containing coding region of Le1637 with a E243 substitution by A243 |
| pBAD-Le1637^E243A^- overlap-F | TACAACGTCAGCGGCGCGATCCTGACCGCGGCG |  |
| pBAD-Le0849-F | ACAGGAGGAATTAACCATGGATGTGCGGCTGGCGGAAACGGG | To amplify a 420-bp fragment containing coding region of Le0849 |
| pBAD-Le0849-R | TTTTGTTCGGGCCCAAGCTTATCGCCCAGCGTCTTGACGA |  |
| pBAD-Le1374-F | ACAGGAGGAATTAACCATGGATATGAACGCATCCACCGACCG | To amplify a 453-bp fragment containing coding region of Le1374 |
| pBAD-Le1374-R | TTTTGTTCGGGCCCAAGCTTCTCGACATAGCGCGCGATGT |  |
| pBAD-Le4706-F | ACAGGAGGAATTAACCATGGATGTGATCGCGGTCGACTCGCC | To amplify a 396-bp fragment containing coding region of Le4706 |
| pBAD-Le4706-R | TTTTGTTCGGGCCCAAGCTTGGCTTGCGGCACGATCAGCT |  |
| pBAD-Le4802-F | ACAGGAGGAATTAACCATGGATATGACGACACTGTGGCGCAT | To amplify a 477-bp fragment containing coding region of Le4802 |
| pBAD-Le4802-R | TTTTGTTCGGGCCCAAGCTTAGGCGCGCGGAACACCACGT |  |
| pBAD-Le4886-F | ACAGGAGGAATTAACCATGGATATGAACCTGTCCGCCAACGA | To amplify a 549-bp fragment containing coding region of Le4886 |
| pBAD-Le4886-R | TTTTGTTCGGGCCCAAGCTTGGCCTGCGGGATGCGCTGTT |  |
| Pull-down assays | | |
| pBBR1-GFP-FLAG-F | GGGAACAAAAGCTGGGTACCGATGAGTAAAGGTGAAGAACT | To amplify a 717-bp fragment containing coding region of GFP with a C-terminal FLAG tag |
| pBBR1-GFP-FLAG-R | GCGGTGGCGGCCGCTCTAGATTTGTAGAGTTCATCCATGC |  |
| pBADGM-GFP-Myc-F | ACAGGAGGAATTAACCATGGATATGAGTAAAGGTGAAGAACT | To amplify a 717-bp fragment containing coding region of GFP with a C-terminal Myc tag |
| pBADGM-GFP-Myc-R | TTTTGTTCGGGCCCAAGCTTTTTGTAGAGTTCATCCATGC |  |
| pBADGM-XAC2246-FLAG-F | ACAGGAGGAATTAACCATGGATATGACGATGAAAAATAAGCC | To amplify a 636-bp fragment containing coding region of GFP with a C-terminal FLAG tag |
| pBADGM-XAC2246-FLAG-R | TTTTGTTCGGGCCCAAGCTTCTATTATCATCACTTATCGTCGTCATCCTTGTAATCGTATTTCGCGGCGTCTT |  |
| pBADGM-XAC2248-FLAG-F | ACAGGAGGAATTAACCATGGATGTGCCGGATCCGGACTCCTG | To amplify a 669-bp fragment containing coding region of GFP with a C-terminal FLAG tag |
| pBADGM-XAC2248-FLAG-R | TTTTGTTCGGGCCCAAGCTTTTATCACTACTACTTATCGTCGTCATCCTTGTAATCTCCGCCACCACC |  |
| pBADGM-Le1636-FLAG-F | ACAGGAGGAATTAACCATGGATATGATGCGTACCGCCGCGAT | To amplify a 738-bp fragment containing coding region of Le1636 with a C-terminal FLAG tag |
| pBADGM-Le1636-FLAG-R | TTTTGTTCGGGCCCAAGCTTCTATTATCATCACTTATCGTCGTCATCCTTGTATCAGCGCCCGCCGTCCTCCA |  |
| pBADGM-Le1636-FLAG-F | ACAGGAGGAATTAACCATGGATATGCCTGTTTCGATCTCCGC | To amplify a 1746-bp fragment containing coding region of Le1638 with a C-terminal FLAG tag |
| pBADGM-Le1638-FLAG-R | TTTTGTTCGGGCCCAAGCTTCTATTATCATCACTTATCGTCGTCATCCTTGTATCAATACCCCGCCGCCTGCC |  |


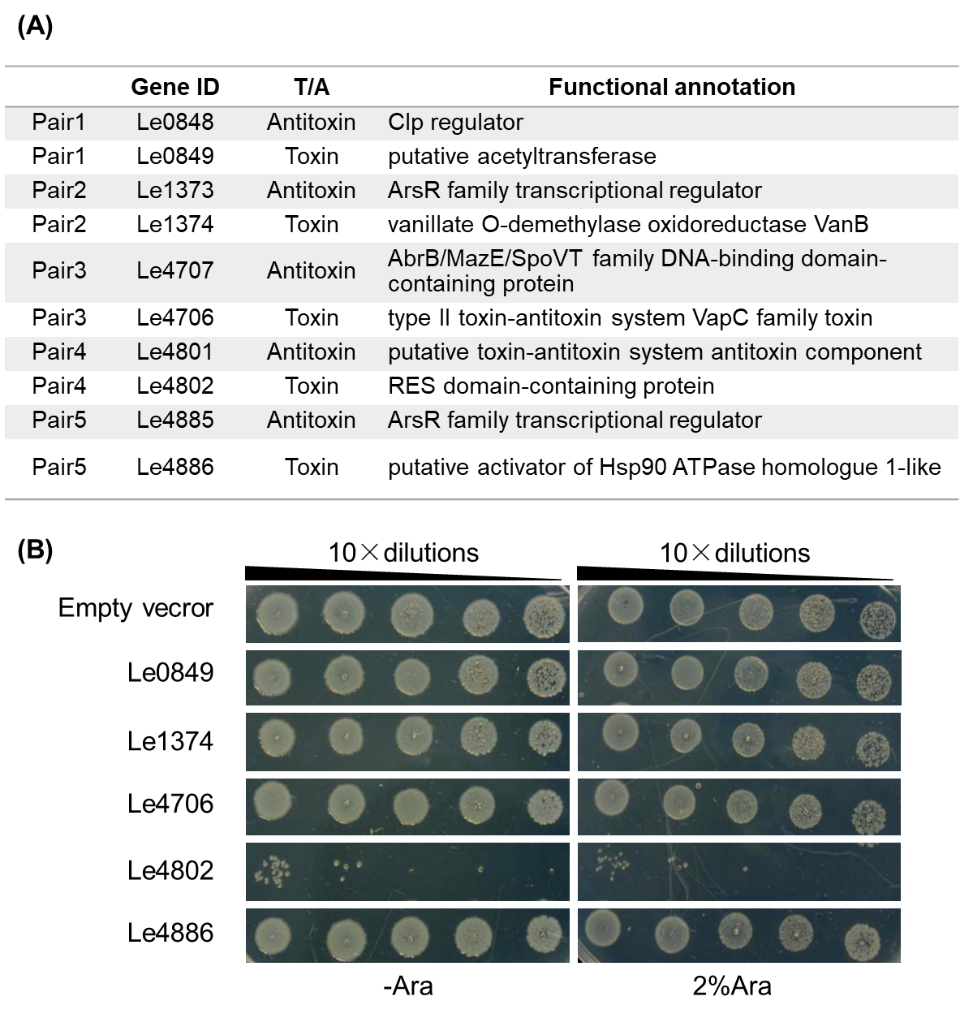


**Figure S1. Prediction and screening toxin proteins of type II TA systems in *Lysobacter enzymogenes* OH11 by the web-based database TADB2**. **(A)** Genomic presence of type II TA system predicted by the publicly accessible database TADB2; **(B)** Screening of predicted toxin proteins of type II TA systems in *E. coli.* Each predicted toxin gene was expressed in *E. coli* BL21 (DE3) under an arabinose-inducible promoter within the pBAD/Myc-HisA vector. *E. coli* cells expressing the toxin genes were streaked onto LB medium supplemented with (+Ara) or without (-Ara) arabinose. Plates were incubated at 37°C. Insufficient bacterial growth on plate containing arabinose revealed that the gene (Le4802) encoded a toxin.

**Figure S2. Sequence alignment of Le1637 with other T4E homologues.**

**Figure S3. Growth monitoring of *E. coli* cells carrying Le1653/Le3870/Le5014**. *E. coli* BL21 (DE3) cultures (OD600 = 0.03) transformed with pBAD-Le1653/Le3870/Le5014 and an empty pBAD vector were induced with 2% L-arabinose at 0 h. Cell growth was recorded hourly at OD600. Mean ± standard deviation of triplicate replicates for each treatment is represented by column. Experiments were repeated at least 3 times on different days.


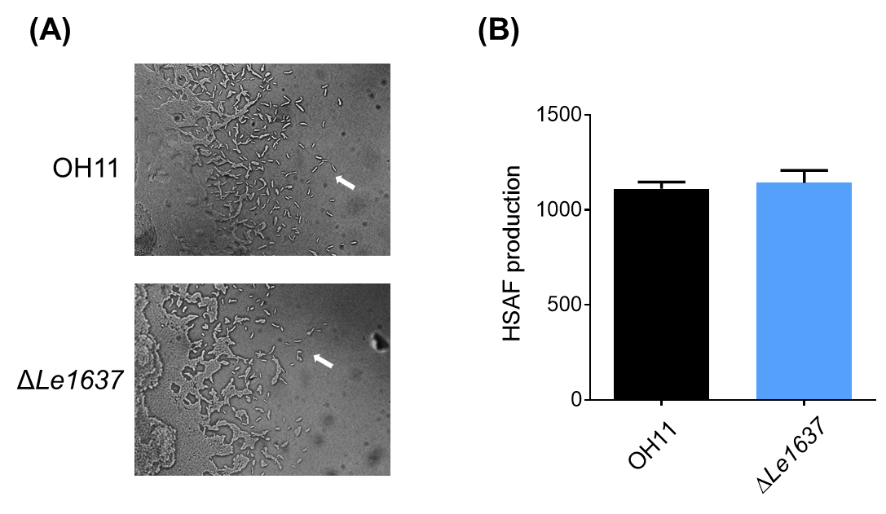


**Figure S4. Mutation of *Le1637* did not impair twitching motility and HSAF production in *L. enzymogenes***. **(A)** Microscopic observation of twitching motility in wild-type OH11 and in-frame *Le1637* deletion mutant. Mobile cells at the colony edge of each tested strain are indicated by arrows, which is a characteristic phenotype of twitching motility in *L. enzymogenes*; (**B)** HSAF production, quantified by high performance liquid chromatography (HPLC).


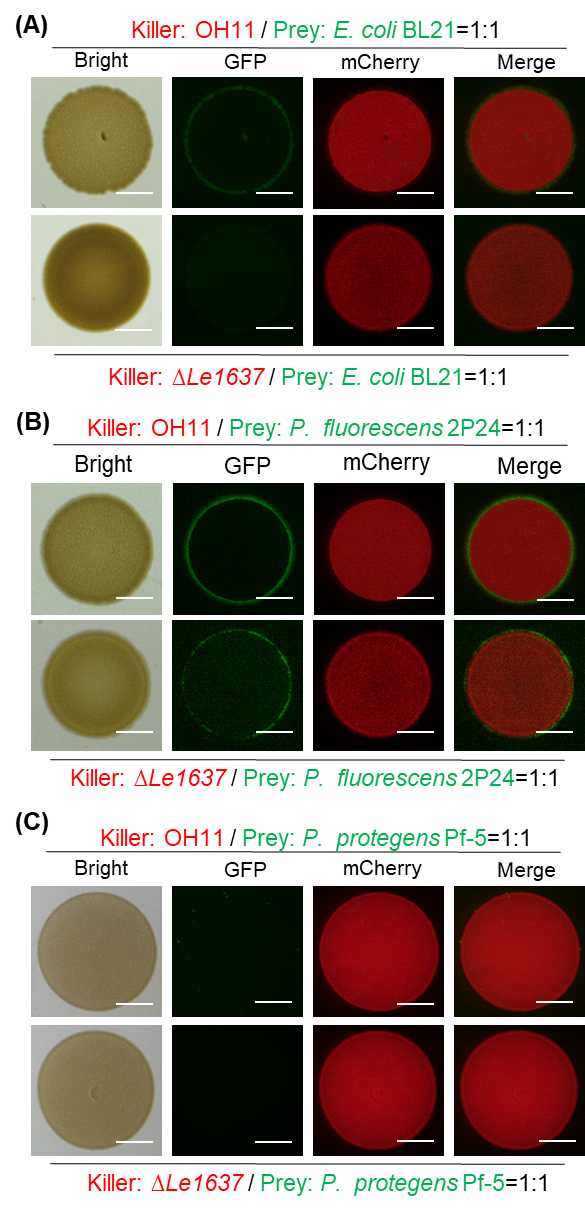


**Figure S5. Le1637 did not play a role in T4SS-mediated bacterial-killing events. (A)-(C)**. Cell-to-cell contact-dependent killing activity of mCherry-labelled OH11 against GFP-labelled *Escherichia coli* BL21 (**A**), *Pseudomonas (P.) fluorescens* 2P24 (**B**), and *P. protegens* Pf-5 (**C**). MCherry-labelled killer strains (OH11 or Δ*Le1637*) were mixed with GFP-labelled competitor strains at a ratio of 1:1 and co-inoculated on agar plates for 24 h, followed by observing the GFP and mCherry fluorescence signals. Bars indicate 2 mm.


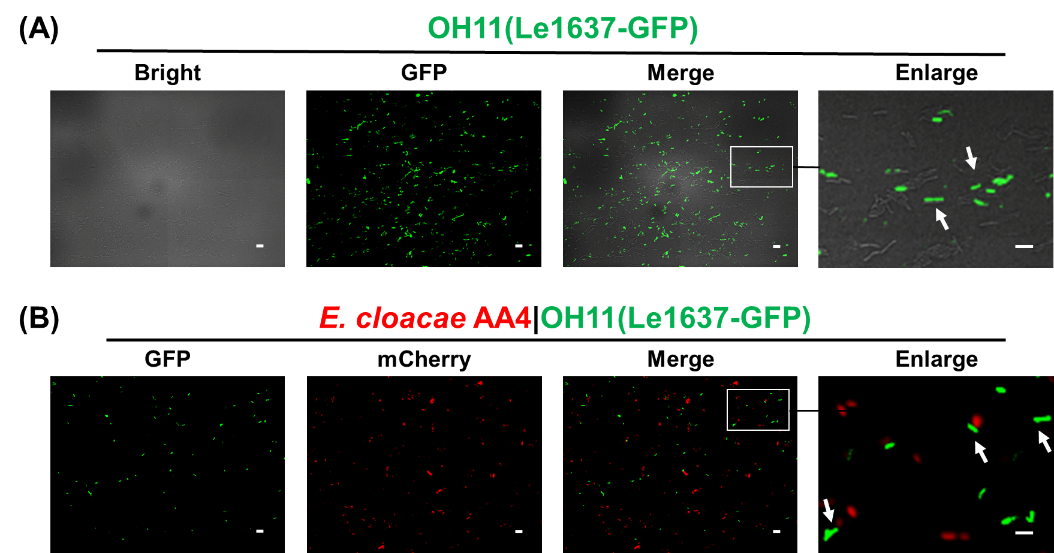


**Figure S6. Visualization of the localization Le1637-GFP in *L. enzymogenes* by fluorescence microscope**. (**A**) Le1637-GFP was distributed throughout the cells when applied to wild-type OH11 monoculture on agar plates was. (**B**) Compared to monoculture, the cellular localization of Le1637-GPF was not altered when OH11 and *Enterobacter cloacae* AA4 were co-cultured on agar plates to mimic their cell-to-cell contact. Bars represent 5 μm.


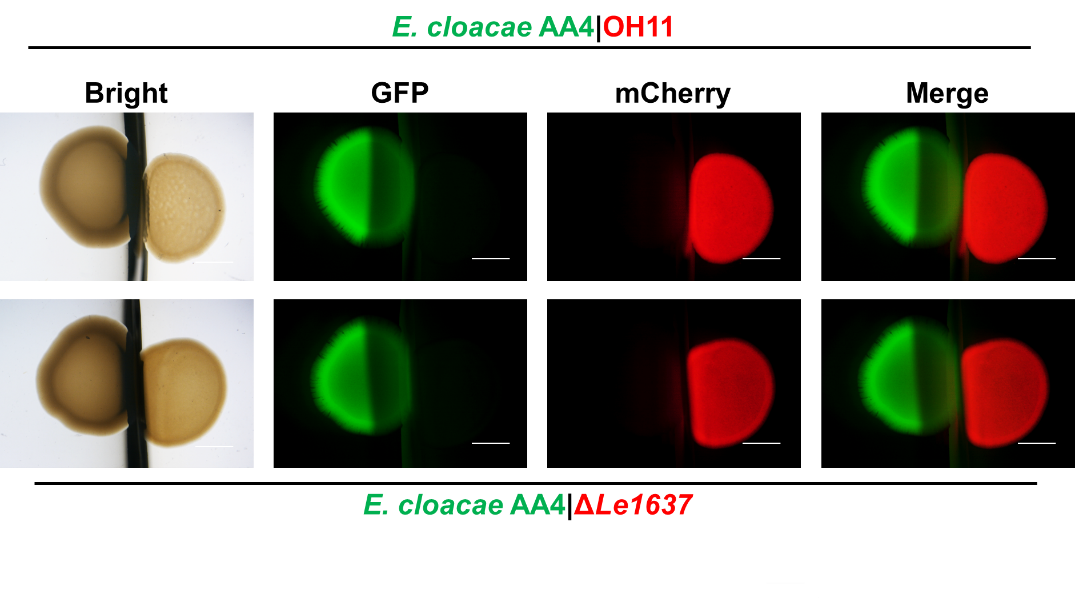


**Figure S7. Killing of *L. enzymogenes* by *Enterobacter cloacae* triggered by cell-to-cell mixing was blocked by filter separation.** MCherry-labelled *L. enzymogenes* OH11 and Δ*Le1637* was co-cultured with each of the GFP-labelled *E. cloacae* AA4 at a ratio of 1:1. A 0.22-μM filter was used to separate the growth of the mCherry-labelled prey (OH11 or Δ*Le1637*) from the GFP-labelled killer (AA4). Fluorescence signals were observed after 24 hours of incubation. Bars represent 2 mm.

**References:**

1 Qian G, Hu B, Jiang Y, Liu F. Identification and characterization of *Lysobacter enzymogenes* as a biological control agent against some fungal pathogens. *Agricultural Sciences in China*. 2009;8:68-75.

2 Shen X, Wang B, Yang N, Zhang L, Shen D, Wu H, et al. *Lysobacter enzymogenes* antagonizes soilborne bacteria using the type IV secretion system. *Environ Microbiol*. 2021;23:4673-4688.

3 Qian G, Wang Y, Liu Y, Xu F, He Y, Du L, et al. *Lysobacter enzymogenes* uses two distinct cell-cell signaling systems for differential regulation of secondary-metabolite biosynthesis and colony morphology. *Appl Environ Microb*. 2013;79:6604-6616.

4 Su Z, Chen H, Wang P, Tombosa S, Du L, Han Y, et al. 4-Hydroxybenzoic acid is a diffusible factor that connects metabolic shikimate pathway to the biosynthesis of a unique antifungal metabolite in *Lysobacter enzymogenes*. *Mol Microbiol*. 2017;104:163-178.

5 Niu B, Paulson JN, Zheng X, Kolter R. Simplified and representative bacterial community of maize roots. *Proc. Natl. Acad. Sci. U.S.A.* 2017;114: E2450-E2459.

6 Kovach ME, Elzer PH, Steven Hill D, Robertson GT, Farris MA, Roop RM, et al. Four new derivatives of the broad-host-range cloning vector pBBR1MCS, carrying different antibiotic-resistance cassettes. *Gene*. 1995;166:175-176.

7 Xu G, Han S, Huo C, Chin K, Chou S, Gomelsky M, et al. Signaling specificity in the c-di-GMP-dependent network regulating antibiotic synthesis in *Lysobacter*. *Nucleic Acids Res*. 2018;46:9276-9288.

8 Wang J, Shen D, Ge C, Du Y, Lin L, Liu J, et al. *Filamentous phytophthora* pathogens deploy effectors to interfere with bacterial growth and motility. *Front Microbiol*. 2020;11: 581511.

9 Ling J, Wang H, Wu P, Li T, Tang Y, Naseer N, et al. Plant nodulation inducers enhance horizontal gene transfer of *Azorhizobium caulinodans* symbiosis island. *Proc. Natl. Acad. Sci. U.S.A.* 2016;113:13875-13880.
